# Cryogenic OrbiSIMS Localizes Semi‐Volatile Molecules in Biological Tissues†

**DOI:** 10.1002/anie.202006881

**Published:** 2020-08-13

**Authors:** Clare L. Newell, Jean‐Luc Vorng, James I. MacRae, Ian S. Gilmore, Alex P. Gould

**Affiliations:** ^1^ Physiology and Metabolism Laboratory The Francis Crick Institute 1 Midland Road London NW1 1AT UK; ^2^ NiCE-MSI National Physical Laboratory Hampton Road Teddington TW11 0LW UK; ^3^ Metabolomics Science Technology Platform The Francis Crick Institute 1 Midland Road London NW1 1AT UK

**Keywords:** analytical methods, biological mass spectrometry imaging, lipids, semi-volatile molecules

## Abstract

OrbiSIMS is a recently developed instrument for label‐free imaging of chemicals with micron spatial resolution and high mass resolution. We report a cryogenic workflow for OrbiSIMS (Cryo‐OrbiSIMS) that improves chemical detection of lipids and other biomolecules in tissues. Cryo‐OrbiSIMS boosts ionization yield and decreases ion‐beam induced fragmentation, greatly improving the detection of biomolecules such as triacylglycerides. It also increases chemical coverage to include molecules with intermediate or high vapor pressures, such as free fatty acids and semi‐volatile organic compounds (SVOCs). We find that Cryo‐OrbiSIMS reveals the hitherto unknown localization patterns of SVOCs with high spatial and chemical resolution in diverse plant, animal, and human tissues. We also show that Cryo‐OrbiSIMS can be combined with genetic analysis to identify enzymes regulating SVOC metabolism. Cryo‐OrbiSIMS is applicable to high resolution imaging of a wide variety of non‐volatile and semi‐volatile molecules across many areas of biomedicine.

## Graphical abstract

Cryogenic OrbiSIMS decreases molecular fragmentation and expands the chemical space that is amenable to mass spectrometry imaging with high spatial and mass resolution. Semi‐volatile and non‐volatile molecules can now be imaged simultaneously in biological tissues.

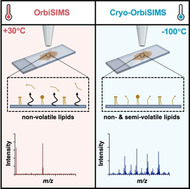

Numerous semi‐volatile organic compounds (SVOCs) such as aldehydes, esters and hydrocarbons are synthesized by plants, humans and other animals. SVOCs perform many different biological functions, with several acting as signals that convey pheromonal communication between individuals of the same or other species.^1^, ^2^, ^3^ Mass spectrometry of headspace vapors, impregnated fibers or solvent extracts has traditionally been used to analyze SVOCs produced by biological tissues.^4^ These methods are very sensitive but do not localize SVOCs in tissues with high spatial resolution. Chemical imaging of hydrocarbons is possible using matrix assisted laser desorption ionization (MALDI) and ultraviolet laser desorption ionization (UV‐LDI) methods.^5^, ^6^ As both techniques are limited by laser spot size, it is desirable to develop higher spatial resolution methods such as secondary ion mass spectrometry (SIMS). SVOCs are, however, challenging to analyze with SIMS as they are volatile under the ultra‐high vacuum required by these instruments, and therefore cannot be imaged. To overcome this limitation, cryogenic temperatures can be used to lower the vapor pressures of SVOCs. Cryogenic workflows that prevent the ice sublimation of frozen‐hydrated samples have been developed for SIMS instruments with a time‐of‐flight detector (ToF‐SIMS).^7^, ^8^ ToF detectors in SIMS instruments have excellent sensitivity and fast data acquisition but they tend not to have sufficient mass resolution to assign peaks with high confidence.

The OrbiSIMS is a new hybrid SIMS instrument that combines ToF and Orbitrap detectors to allow chemical imaging with micron spatial resolution.^9^ The Orbitrap analyzer provides high mass resolution (>240 000 at *m*/*z* 200) with <2 ppm mass accuracy, enabling peaks to be assigned with a higher confidence, albeit at a slower data acquisition rate than for a ToF analyzer. Hence, the OrbiSIMS allows fast image acquisition and high sensitivity using the ToF analyzer to be combined with high mass resolution via the Orbitrap analyzer. We therefore set out to develop a cryogenic workflow for OrbiSIMS that would allow SVOCs to be imaged with high chemical and spatial resolution (Experimental Section). As a test case, we chose hydrocarbons because they are biologically important SVOCs but many are volatile under high vacuum at ambient temperature (Supplementary Figure 1). Hydrocarbons are extensively fragmented by many commonly used ion sources,^10^ making it difficult to identify the parent ions. As hydrocarbon adducts have not previously been reported in SIMS, we first analyzed their chemical composition via Cryo‐OrbiSIMS in positive polarity spectrometry mode—using either the 20 keV argon gas cluster ion beam (GCIB) or the 60 keV Bi_3_^++^ liquid metal ion gun (LMIG). Analysis of (*Z*)‐9‐tricosene, an insect pheromone hydrocarbon,^11^ showed that the 20 keV Ar_3500_^+^ GCIB tends to generate [*M*+N]^+^ ions, which may be formed from direct interactions with nitrogen gas in the instrument or via multiple hydrogen loss from [*M*+NH_4_]^+^ adducts (Supplementary Figure 2). In contrast, the 60 keV Bi_3_^++^ LMIG primarily yields [*M*−2 H]^+.^ ions, as previously reported for GC‐MS,^12^ but leads to greater fragmentation of tricosene. GCIB analysis of an American Society for Testing and Material (ASTM) reference gas oil, a mix including a range of C10:0 to C52:0 alkanes, also showed abundant [*M*+N]^+^ ions (Supplementary Figure 3). Hydrocarbon peak assignments were validated by comparing MS and MS/MS spectra from the OrbiSIMS Orbitrap with those from direct infusion HESI‐Orbitrap and GC‐MS (Supplementary Figure 4). These findings show that a cryogenic OrbiSIMS workflow reliably detects hydrocarbons and they also identify the major adducts formed.

Chemical imaging of human latent fingerprints has emerged as an important area of forensic and biomedical science. Several imaging technologies including ToF‐SIMS have previously been used to localize non‐volatile drugs, poisons, antibiotics and other molecules to the ridges and sweat pores of fingerprints.^13^, ^14^, ^15^ Comparison of cryo‐ and ambient OrbiSIMS Orbitrap spectra showed the presence of many peaks at −110 °C that are not observed at 30 °C (Figure 1). Orbitrap putative peak assignments indicate that many such peaks correspond to fatty acids and their derivatives as well as triacylglycerides (TAGs) and phosphoinositol‐ceramides (PI‐Cer) (Supplementary Table 1). Fatty acids are known to be abundant in latent fingerprints but have not been robustly detected in previous fingerprint studies using SIMS at ambient temperature, presumably because they are volatile under these high‐vacuum conditions (Supplementary Figure 1). Consistent with this, signals from C16:1 and C16:0 were reproducibly detected using OrbiSIMS in the cryogenic but not in the ambient mode. It is therefore surprising that previous studies using SIMS at ambient temperature have reported free fatty acids in other tissues.^16^, ^17^ One possible explanation is that the appearance of free fatty acids at ambient temperature results from beam‐induced fragmentation of TAGs and/or other high molecular weight lipids that are not themselves volatile under ultra‐high vacuum. In line with this, 20 keV Ar_3500_^+^‐Orbitrap OrbiSIMS analysis of latent fingerprints at ambient, compared to cryogenic, temperature shows decreased TAG and increased diacylglyceride signals (Supplementary Table 1). Molecular fragmentation occurs because of excess internal energy. This can be decreased by either decreasing the vibrational energy of the molecule or the amount of energy transferred via a series of collisions from the incoming primary ion.^18^ We therefore investigated how fragmentation of a TAG standard, triolein, in the OrbiSIMS instrument varies as a function of sample temperature and also the energy per atom of the 20 keV Ar_*n*_^+^ primary ion beam. Triolein is detected as [*M*+Na]^+^ at *m*/*z* 907.77 and its fragmentation can be measured by the ratio of this molecular ion to the total ion counts in the spectrum. At cryogenic temperatures, triolein fragmentation is decreased for all energies per atom with an order of magnitude decrease at 5.7 eV per atom (*n*=3500) (Supplementary Figure 5 a,b). In addition, cryogenic temperatures increase the absolute yield of the [*M*+Na]^+^ molecular ion by a factor of ca. 4.5 (Supplementary Figure 5 c and Supplementary Table 1). These findings indicate that, in contrast to lowering the energy per atom of the primary ion, cryogenic analysis temperatures decrease fragmentation without sacrificing ionization yield. We conclude that cryogenic sample analysis, together with low energy per atom of the primary ion, improves SIMS detection of biologically important lipids in their intact state by an order of magnitude.

**Figure 1:**
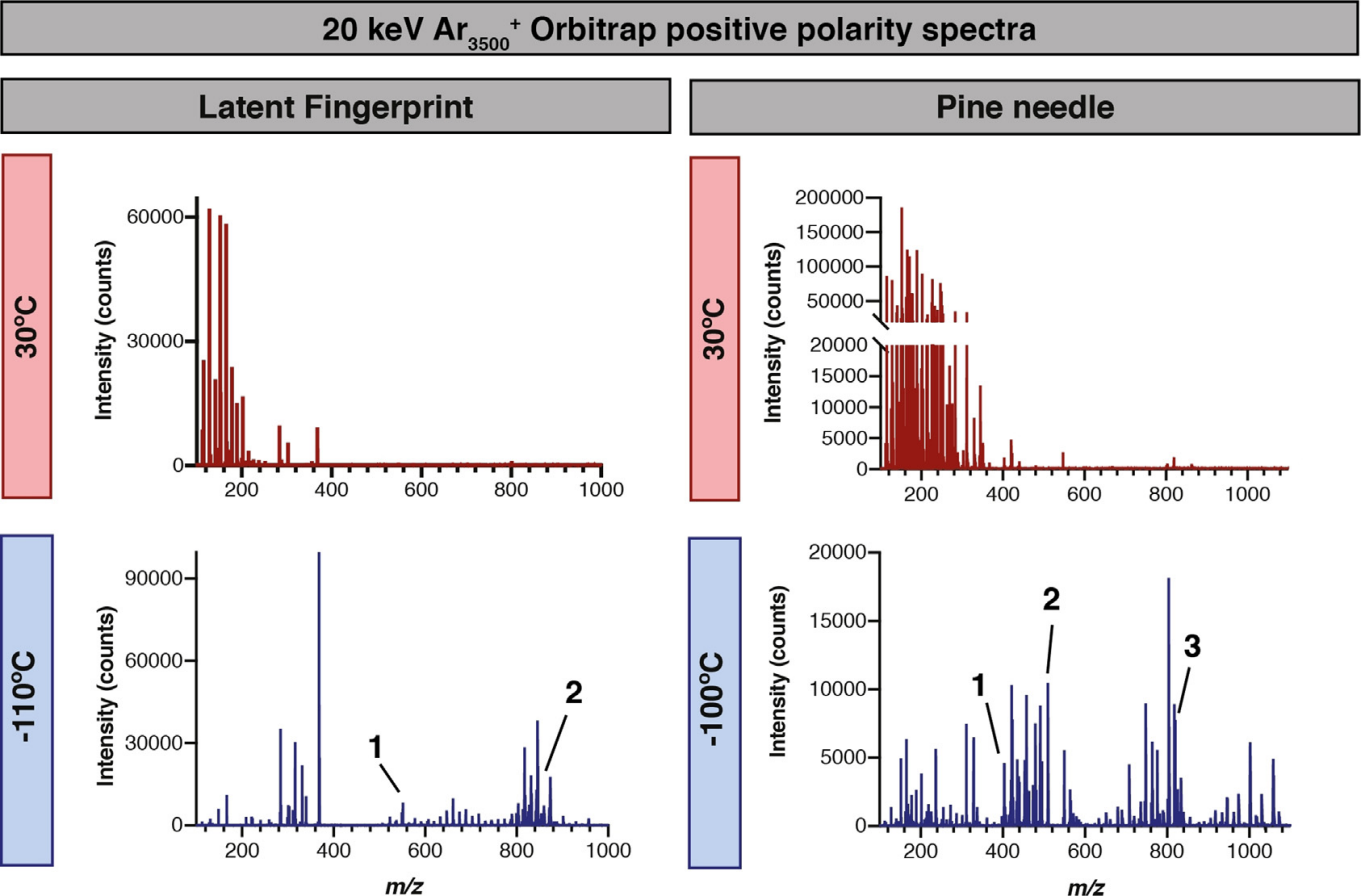
**Cryo‐OrbiSIMS decreases molecular fragmentation and increases chemical coverage**. Comparison of OrbiSIMS spectra of a latent fingerprint and a pine needle with positive polarity at cryogenic (−100 or −110 °C) and ambient (30 °C) temperatures using a 20 keV Ar_3500_^+^ GCIB with a ca. 3 μm spot size from a 400×400 μm field of view and 20×20 pixel resolution. Many more features are detected with cryogenic analysis, including putatively annotated intact triglycerides and semi‐volatile hydrocarbons. Fingerprint putative annotations: (1) *m*/*z* 551.5041, [C_35_H_67_O_4_]^+^ with mass deviation δ 1.4 ppm, C32:0 diacylglyceride adduct [*M*+H−H_2_O]^+^ (2) *m*/*z* 855.741, [C_53_H_100_O_6_Na]^+^ with mass deviation δ−0.25 ppm, C50:1 triacylglyceride adduct [*M*+Na]^+^. Pine needle putative annotations: (1) *m*/*z* 403.4295, [C_29_H_55_]^+^ with mass deviation δ−0.7 ppm, C29:0 hydrocarbon adduct [*M*−5 H]^+^ also identified in GC‐MS as [*M*]^+.^ alongside a commercial standard (Supplementary Table 1) (2) *m*/*z* 509.4561, [C_32_H_61_O_4_]^+^ with mass deviation δ−0.6 ppm, C29:0 diacylglyceride adduct [*M*+H−H_2_O]^+^. (3) *m*/*z* 819.6379, [C_48_H_88_N_2_O_6_P]^+^ with mass deviation δ 0.6 ppm, C40:5 phosphatidylcholine adduct [*M*+NH_4_−H_2_O]^+^. A full list of putative peak annotations and validation methods is available in Supplementary Table 1.

To determine the spatial localizations of lipids and other molecules in latent fingerprints, Cryo‐OrbiSIMS was used in ToF MS imaging mode. This revealed that [Na_3_N_2_O_2_]^+^ (*m*/*z* 128.97) specifically localizes to the vicinity of sweat (eccrine) pores, whereas [NaP_2_O_6_]^+^ (*m*/*z* 180.91) is in a complementary pattern, largely excluded from sweat pores (Figure 2 a). Strong lipid signals were detected close to sweat pores for hexadecenoic acid (C16:1, corresponding Orbitrap *m*/*z* 299.1956, likely representing sapienic acid) and palmitic acid (C16:0, corresponding Orbitrap *m*/*z* 301.2113) (Figure 2 a). These two fatty acids were observed in both the 30 keV Bi_3_^+^‐ToF and in the 20 keV Ar_3500_^+^‐Orbitrap spectra under cryogenic but not ambient analysis temperature. Hence, Cryo‐OrbiSIMS imaging of fingerprints identifies lipids and other molecules specific for sweat pore versus inter‐pore regions. Such localized markers may provide useful chemical signatures for forensic and biomedical research.

**Figure 2:**
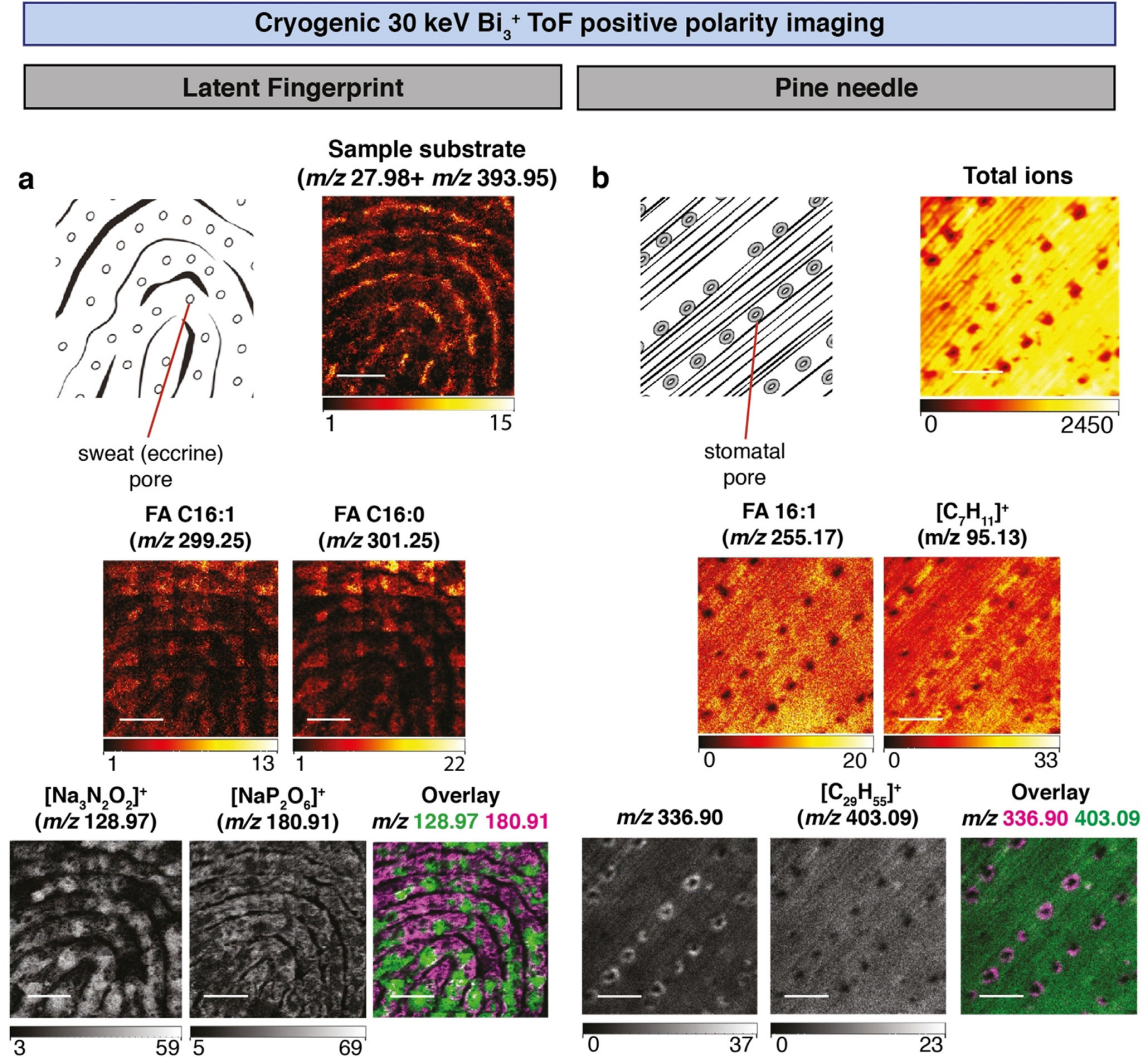
**Localization of semi‐volatile molecules in pine needles and latent fingerprints using Cryo‐OrbiSIMS**. a) Positive polarity imaging of a latent fingerprint (diagram indicates eccrine pores) using a 30 keV Bi_3_^+^ LMIG with an approximate spot size of 0.5 μm and ToF analyzer of a 2.5×2.5 mm field of view with 5120×5120 pixel resolution, binned to 1280×1280 pixels. Sample substrate refers to the sum of *m*/*z* 27.98 (Si^+^) and *m*/*z* 393.95 (Au_2_^+^), components of the gold‐coated silicon wafer. Other mass images show four compounds detected at cryogenic but not at ambient temperatures. Fingerprint putative annotations: *m*/*z* 299.1956 (detected in Orbitrap and ToF spectra), [C_16_H_29_O_2_Na_2_]^+^ (Orbitrap mass deviation δ−0.5 ppm), hexadecenoic acid (FA 16:1) adduct [*M*+2 Na−H]^+^. *m*/*z* 301.2113 (detected in Orbitrap and ToF spectra), [C_16_H_31_O_2_Na_2_]^+^ (Orbitrap mass deviation δ−0.4 ppm), palmitic acid (FA 16:0) adduct [*M*+2 Na−H]^+^. *m*/*z* 128.97 (detected only in ToF spectra), [Na_3_N_2_O_2_]^+^ (ToF mass deviation δ 30.6 ppm) localizes close to sweat pores. *m*/*z* 180.91 (detected only in ToF spectra), [NaP_2_O_6_]^+^ (ToF mass deviation δ 31.2 ppm) localizes in a pattern excluding sweat pores. Scale bars represent 0.625 mm. b) Positive polarity imaging of a pine needle (*Pinus nigra*, diagram indicates stomatal pores*)* using a 30 keV Bi_3_^+^ LMIG with an approximate spot size of 0.5 μm and ToF analyzer of a 500×500 μm field of view with 1024×1024 pixel resolution, binned to 256×256 pixels. Total ion count image shows position of stomatal pores and *m*/*z* 366.90 provides a diagnostic ion for the guard cells surrounding the pores and is detected at cryogenic but not ambient temperature. Pine needle putative annotations: *m*/*z* 255.17, [C_18_H_35_O_2_]^+^, linoleic acid (FA 18:2) adduct [*M*+H]^+^ is detected at both cryogenic and ambient temperatures and with the Orbitrap analyzer (*m*/*z* 255.2317 and mass deviation δ−0.7 ppm). *m*/*z* 95.12, [C_7_H_11_]^+^, likely represents a volatile hydrocarbon fragment and is also detected by EI‐GC‐MS/MS and OrbiSIMS MS/MS analysis of hydrocarbon standards (Supplementary Figure 4). *m*/*z* 403.09, [C_29_H_55_]^+^, is a C29:0 hydrocarbon adduct [*M*−5 H]^+^ also detected with the Orbitrap analyzer (*m*/*z* 403.4295 and mass deviation δ−0.7 ppm) and in the [*M*]^+.^ form via GC‐MS alongside a commercial standard (Supplementary Table 1). The [*M*−5 H]^+^ ion is not the most abundant hydrocarbon adduct but is shown due to clear separation from surrounding peaks. Scale bars represent 125 μm. In both panels, the intensity ranges (in counts) are represented beneath each mass image.

Hydrocarbons, particularly long‐chain alkanes, are a major component of the cuticular lipid barrier to water loss in both plants and insects.^11^, ^19^, ^20^, ^21^ We used Cryo‐OrbiSIMS to analyze the cuticle on the surface of pine needles of *Pinus nigra* revealing that signal from the ion *m*/*z* 336.90 provides a specific chemical marker for the guard cells of the stomatal pores (Figure 2 b). This contrasts with more spatially uniform epicuticular signals for putative hexadecenoic acid (*m*/*z* 255.17) and the volatile hydrocarbon nonacosane (*m*/*z* 403.09), which we validated with GC‐MS alongside a commercial standard (Supplementary Table 1). Importantly, these three molecular ions could be reliably detected on the surface of pine needles using cryogenic but not ambient temperatures. Analysis of a variety of different plant leaf and fruit samples illustrates the broad diversity of hydrocarbons and other SVOCs that can be detected cryogenically (Supplementary Table 1). We also utilized the depth‐profiling mode of the Cryo‐OrbiSIMS in positive polarity to demonstrate that the semi‐volatile hydrocarbon octacosane is enriched at the surface of the cuticle of the gooseberry fruit (Supplementary Figure 6 a). Depth profiling of the fruit in negative polarity mode also detected a uniformly distributed putative wax ester, and a free fatty acid enriched in a layer below the surface of the fruit cuticle (Supplementary Figure 6 a). The fingerprint and plant cuticle analyses together demonstrate that the new cryogenic workflow expands the chemical space amenable to SIMS imaging to encompass hydrocarbons and other SVOCs, as well as molecules with intermediate vapor pressures like free fatty acids.

The lipid layer that coats the cuticle of the fruit fly *Drosophila melanogaster* is known to be rich in hydrocarbons, wax esters and other lipids.^11^ We set out to test whether cryo‐OrbiSIMS could reveal new aspects of the biology of this already well characterized system. Orbitrap spectra of abdominal male *Drosophila* cuticle showed a dramatic increase in the number of features that could be detected via cryogenic rather than ambient OrbiSIMS (Figure 3 a). We verified that many of the Cryo‐OrbiSIMS peaks correspond to alkane and alkene hydrocarbons, examples of SVOCs that could also be detected in cuticular hexane extracts via GC‐MS (Supplementary Figure 7). Cuticular hydrocarbon synthesis occurs in cells called oenocytes and requires *Cyp4g1*, a gene encoding a P450 oxidative decarbonylase.^22^, ^23^ In agreement with a previous study,^22^ Orbitrap spectra revealed that oenocyte‐specific overexpression of Cyp4g1 mRNA is not sufficient to significantly alter the amounts of cuticular hydrocarbons (Supplementary Figure 8). In contrast, oenocyte‐specific knockdown of *Cyp4g1* (*Cyp4g1* RNAi) resulted in a dramatic decrease in alkanes and alkenes, including tricosene (Figure 3 b). Cryo‐OrbiSIMS is therefore a promising new analytical tool for studying the genetic regulation of metabolism with high spatial and chemical resolution.

**Figure 3:**
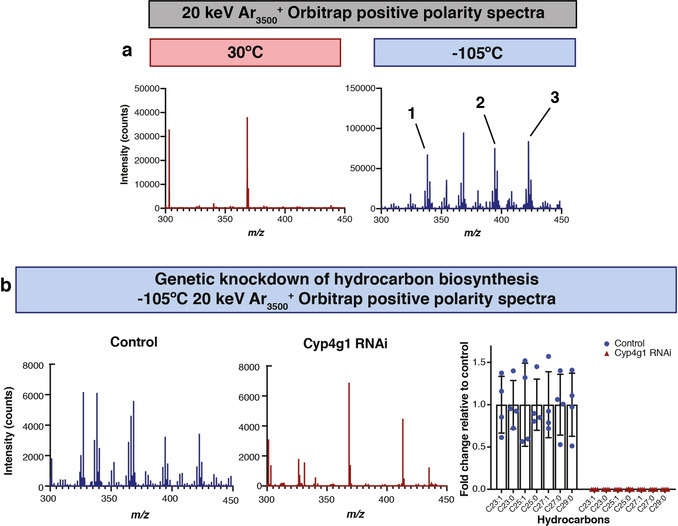
**Metabolic regulation of hydrocarbons on the male** ***Drosophila*** **abdominal cuticle**. b) OrbiSIMS positive polarity spectra using a 20 keV Ar_3500_^+^ GCIB with a spot size of ca. 3 μm highlights that many more features are detected at cryogenic (−105 °C) than at ambient (30 °C) temperatures. Spectra were taken from a field of view of 400×400 μm with 20×20 pixel resolution. Putative annotations: (1) *m*/*z* 338.378, [C_23_H_48_N]^+^ with mass deviation δ 1.43 ppm, C23:0 hydrocarbon adduct [*M*+N]^+^, (2) *m*/*z* 394.440, [C_27_H_56_N]^+^ with mass deviation δ 0.99 ppm, C27:0 hydrocarbon adduct [*M*+N]^+^, (3) *m*/*z* 422.472, [C_29_H_60_N]^+^ with mass deviation δ 1.04 ppm, C29:0 hydrocarbon adduct [*M*+N]^+^. c) Cryo‐OrbiSIMS Orbitrap positive polarity spectra using a 20 keV Ar_3500_^+^ GCIB with a spot size of ca. 3 μm show that cuticular hydrocarbon signals are abundant in genetic control animals (control) but strongly decreased in response to RNAi knockdown of a hydrocarbon biosynthetic enzyme (*Cyp4g1 RNAi*)—see **Experimental Section**. Graph shows fold changes of C23 to C29 hydrocarbons in *Cyp4g1 RNAi* animals versus controls. Bars represent mean, error bars are standard deviation. Spectra were taken from a field of view of 400×400 μm with 20×20 pixel resolution.

Utilizing Cryo‐OrbiSIMS in imaging mode showed that the strong signals from male *Drosophila* hydrocarbons such as tricosane (*m*/*z* 323.27) and a [C_7_H_11_]^+^ (*m*/*z* 95.07) fragment are detected across the cuticle of the abdomen and also the wing (Figure 4 b and Supplementary Figure 9 a). Depth profiling of tricosene and pentacosene shows that, although hydrocarbons are broadly distributed across the abdominal and wing cuticle, they are nevertheless restricted to its surface, known as the epicuticle (Supplementary Figure 6 b,c). The observation that hydrocarbons are rather uniform across the surface of the wing is intriguing given that the cells that synthesize them (oenocytes) are restricted to the abdomen. This indicates that *Drosophila* can efficiently spread their hydrocarbons from one body part to another, likely via a specific grooming behaviour.

**Figure 4:**
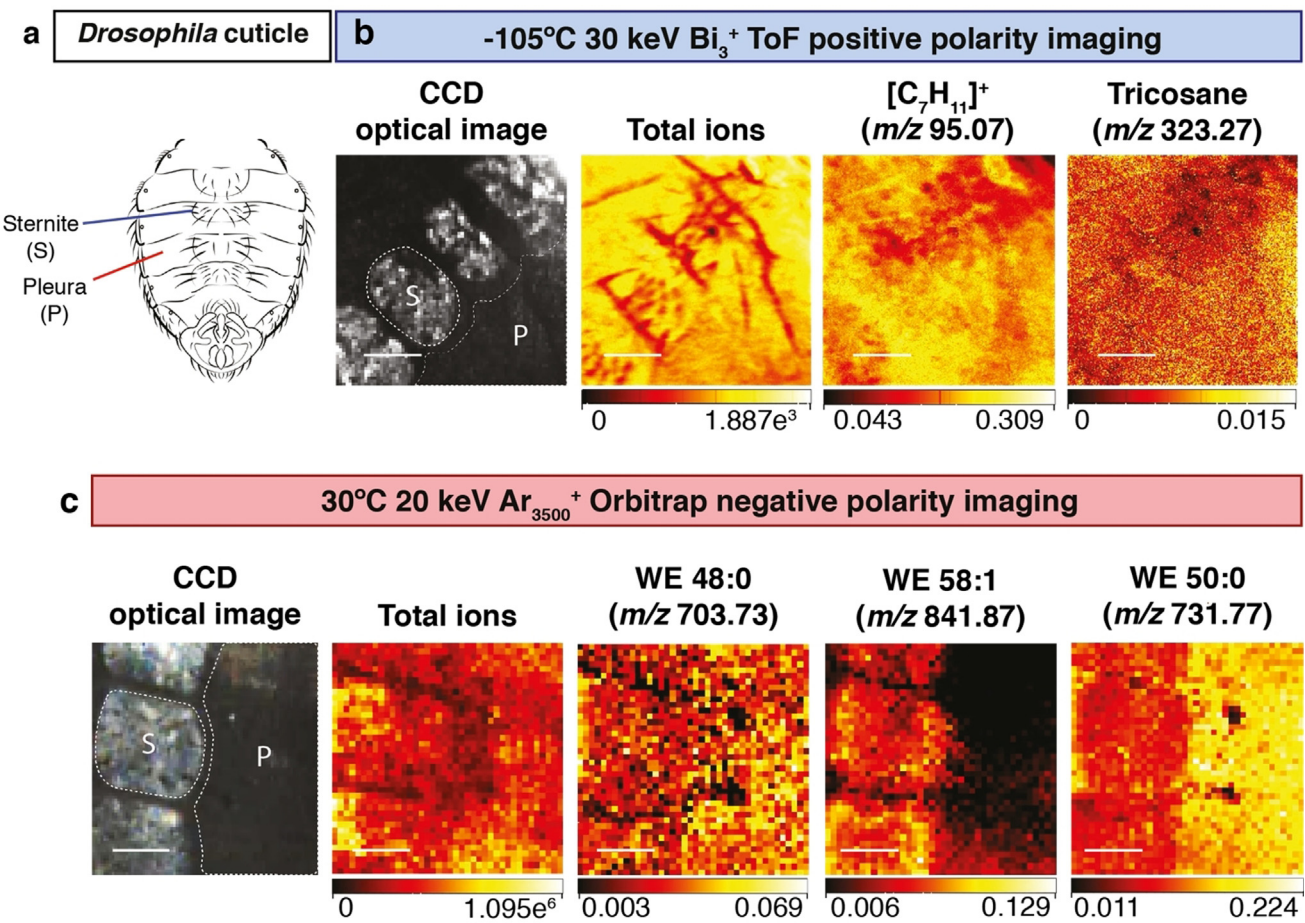
Restricted localization of wax esters on the male *Drosophila* abdominal cuticle. a) The cuticle of the male *Drosophila* abdomen, indicating subdivision into bristle‐bearing ventral plates (sternites) and a bristle‐free lateral region (pleura). b) Cryogenic OrbiSIMS ToF imaging in positive polarity using a 30 keV Bi_3_^+^ LMIG with a spot size of ca. 0.5 μm for a 500×500 μm field of view with 1024×1024 pixel resolution, binned to 256×256 pixels. The charge coupled device (CCD) shows the optical image of the acquired area, with sternites and pleura visible and the corresponding total ion counts at each pixel are shown. Abundant cuticular hydrocarbons are widely distributed across the sternites and pleura of the cuticle. Putative annotations: *m*/*z* 323.27, [C_23_H_47_]^+^, tricosane adduct [*M*−H]^+^, also detected via GC‐MS as [*M*]^+.^ and confirmed with an analytical standard. *m*/*z* 95.07, [C_7_H_11_]^+^, hydrocarbon fragment also detected in EI‐GC/MS and OrbiSIMS Orbitrap MS/MS spectra of hydrocarbon standards (Supplementary Figure 5). Scale bars represent 125 μm c. Ambient OrbiSIMS Orbitrap imaging in negative polarity using a 20 keV Ar_3500_^+^ GCIB with a spot size of ca. 3 μm of a 400×400 μm field of view with 40×40 pixels. The charge coupled device (CCD) shows the optical image of the acquired area, with sternites and pleura visible and corresponding total intensities at each pixel are also shown. Putative annotations: *m*/*z* 703.7341, [C_48_H_95_O_2_]^−^ (mass deviation δ 0.5 ppm), C48:0 saturated wax ester (WE 48:0) near‐uniform across sternites and pleura. *m*/*z* 841.8745, [C_58_H_113_O_2_]^−^ (mass deviation δ−0.1 ppm), C58:1 unsaturated wax ester (WE 58:1) enriched on sternites. *m*/*z* 731.7656, [C_50_H_99_O_2_]^−^ (mass deviation δ 0.7 ppm), C50:0 saturated wax ester (WE 50:0) enriched on pleura. Scale bars represent 100 μm.

OrbiSIMS imaging also revealed that signals from some *Drosophila* putative wax esters are near uniform whereas others are enriched specifically in the pleura (hard plates) or in the sternites (flexible regions) of the abdominal cuticle (Figure 4 a,c). The finding that wax esters, unlike hydrocarbons, are distributed in localized patterns on the cuticle also extends to the wing. For example, the putative wax esters C28:0 and C50:0 are, respectively enriched or depleted at the L3 wing vein (Supplementary Figure 9 b). These non‐uniform localizations suggest that wax esters may define zones of the cuticle with different barrier properties.

Together, the results of this study demonstrate that cryogenic analysis provides two significant analytical advantages for the OrbiSIMS platform. First, it increases the sensitivity of detection for intact biomolecules such as TAGs. And second, it extends chemical coverage to include SVOCs and other intermediate or high vapor pressure molecules. Combining soft ionization with cryogenic analysis conditions has revealed unexpected localization patterns for several biological SVOCs. We anticipate that this SIMS advance will have a broad range of applications in metabolic and pharmaceutical research, forensic science and also in the analysis of environmental pollutants such as polycyclic aromatic hydrocarbons.

## Conflict of interest

The authors declare no conflict of interest.

## Supporting information

As a service to our authors and readers, this journal provides supporting information supplied by the authors. Such materials are peer reviewed and may be re‐organized for online delivery, but are not copy‐edited or typeset. Technical support issues arising from supporting information (other than missing files) should be addressed to the authors.

Supplementary

Supplementary

## References

[1] S. D. Liberles , Annu. Rev. Physiol. 2014, 76, 151–175. 23988175 10.1146/annurev-physiol-021113-170334 PMC4310675

[2] J. Y. Yew , H. Chung , Prog. Lipid Res. 2015, 59, 88–105. 26080085 10.1016/j.plipres.2015.06.001

[3] H. Xu , T. C. J. Turlings , Trends Plant Sci. 2018, 23, 100–111. 29229187 10.1016/j.tplants.2017.11.004

[4] L. Lucattini , G. Poma , A. Covaci , J. de Boer , M. H. Lamoree , P. E. G. Leonards , Chemosphere 2018, 201, 466–482. 29529574 10.1016/j.chemosphere.2018.02.161

[5] V. Vrkoslav , A. Muck , J. Cvačka , A. Svatoš , J. Am. Soc. Mass Spectrom. 2010, 21, 220–231. 19910210 10.1016/j.jasms.2009.10.003

[6] J. Y. Yew , J. Soltwisch , A. Pirkl , K. Dreisewerd , J. Am. Soc. Mass Spectrom. 2011, 22, 1273–1284. 21953110 10.1007/s13361-011-0110-3

[7] K. Kuroda , T. Fujiwara , T. Imai , R. Takama , K. Saito , Y. Matsushita , K. Fukushima , Surf. Interface Anal. 2013, 45, 215–219.

[8] M. Dickinson , P. J. Heard , J. H. A. Barker , A. C. Lewis , D. Mallard , G. C. Allen , Appl. Surf. Sci. 2006, 252, 6793–6796.

[9] M. K. Passarelli , A. Pirkl , R. Moellers , D. Grinfeld , F. Kollmer , R. Havelund , C. F. Newman , P. S. Marshall , H. Arlinghaus , M. R. Alexander , A. West , S. Horning , E. Niehuis , A. Makarov , C. T. Dollery , I. S. Gilmore , Nat. Methods 2017, 14, 1175–1183. 29131162 10.1038/nmeth.4504

[10] S. G. Roussis , W. P. Fitzgerald , A. S. Cameron , Rapid Commun. Mass Spectrom. 1998, 12, 373–381.

[11] G. J. Blomquist , A.-G. Bagneres , Insect hydrocarbons : biology, biochemistry, and chemical ecology, Cambridge University Press, Cambridge, 2010.

[12] F. McLafferty , F. Turecek , Interpretation of Mass Spectra, 4 ed., University Science Books, Sausalito, 1993.

[13] D. R. Ifa , N. E. Manicke , A. L. Dill , R. G. Cooks , Science 2008, 321, 805. 18687956 10.1126/science.1157199

[14] P. Hazarika , D. A. Russell , Angew. Chem. Int. Ed. 2012, 51, 3524–3531; 10.1002/anie.201104313 22461202

[15] L. Cai , M. C. Xia , Z. Wang , Y. B. Zhao , Z. Li , S. Zhang , X. Zhang , Anal. Chem. 2017, 89, 8372–8376. 28700825 10.1021/acs.analchem.7b01629

[16] K. Richter , H. Nygren , P. Malmberg , B. Hagenhoff , Microsc. Res. Tech. 2007, 70, 640–647. 17393479 10.1002/jemt.20450

[17] M. Brulet , A. Seyer , A. Edelman , A. Brunelle , J. Fritsch , M. Ollero , O. Laprévote , J. Lipid Res. 2010, 51, 3034–3045. 20616379 10.1194/jlr.M008870 PMC2936750

[18] T. Fu , S. Della-Negra , D. Touboul , A. Brunelle , J. Am. Soc. Mass Spectrom. 2019, 30, 321–328. 30421360 10.1007/s13361-018-2090-z

[19] L. Kunst , A. L. Samuels , Prog. Lipid Res. 2003, 42, 51–80. 12467640 10.1016/s0163-7827(02)00045-0

[20] S. B. Lee , M. C. Suh , Plant Cell Rep. 2015, 34, 557–572. 25693495 10.1007/s00299-015-1772-2

[21] J.-F. Ferveur , Behav. Genet. 2005, 35, 279. 15864443 10.1007/s10519-005-3220-5

[22] Y. Qiu , C. Tittiger , C. Wicker-Thomas , G. Le Goff , S. Young , E. Wajnberg , T. Fricaux , N. Taquet , G. J. Blomquist , R. Feyereisen , Proc. Natl. Acad. Sci. USA 2012, 109, 14858–14863. 22927409 10.1073/pnas.1208650109 PMC3443174

[23] R. Makki , E. Cinnamon , A. P. Gould , Annu. Rev. Entomol. 2014, 59, 405–425. 24397521 10.1146/annurev-ento-011613-162056 PMC7613053

