# Supplementary material for: Cryogenic OrbiSIMS Localizes Semi‐Volatile Molecules in Biological Tissues
Source: Angew Chem Int Ed Engl. 2020 Aug 13;59(41):18194–200. doi: 10.1002/anie.202006881 (PMC7589292; doi:10.1002/anie.202006881)
Supplement: Supplementary file 1 — Supplementary [file ANIE-59-18194-s001.pdf]

Angewandte Chemie
Eine Zeitschrift der Gesellschaft Deutscher Chemiker

Supporting Information

Cryogenic OrbiSIMS Localizes Semi-Volatile Molecules in Biological Tissues**

Clare L. Newell, Jean-Luc Vorng, James I. MacRae, Ian S. Gilmore, and Alex P. Gould**

anie_202006881_sm_miscellaneous_information.pdf
anie_202006881_sm_Table1.xlsx

Author Contributions

I.S.G and A.P.G. conceived the study and acquired funding. C.L.N acquired OrbiSIMS, ToF-SIMS, Orbitrap direct infusion and GC-MS data. C.L.N, J.-L.V. and I.S.G. developed the cryo-analysis protocols. I.S.G. created the OrbiSIMS concept and cryo-sample handling design concept. C.L.N. and A.P.G. designed the genetics and selected the biological samples to be analyzed. All authors designed OrbiSIMS experiments. C.L.N., J.-L.V., and I.S.G. designed ToF-SIMS experiments. C.L.N. and J.I.M. designed the Orbitrap direct infusion experiment. C.L.N., J.I.M. and A.P.G. designed GC-MS and LC-MS experiments. C.L.N. analyzed and interpreted the data, with additional interpretation from J.-L.V., J.I.M., I.S.G. and A.P.G. C.L.N., I.S.G. and A.P.G. wrote the manuscript. All authors commented on and approved the manuscript.

Antoine Equation: $$\log_{10}(P) = A - \left(\frac{B}{(T + C)}\right)$$

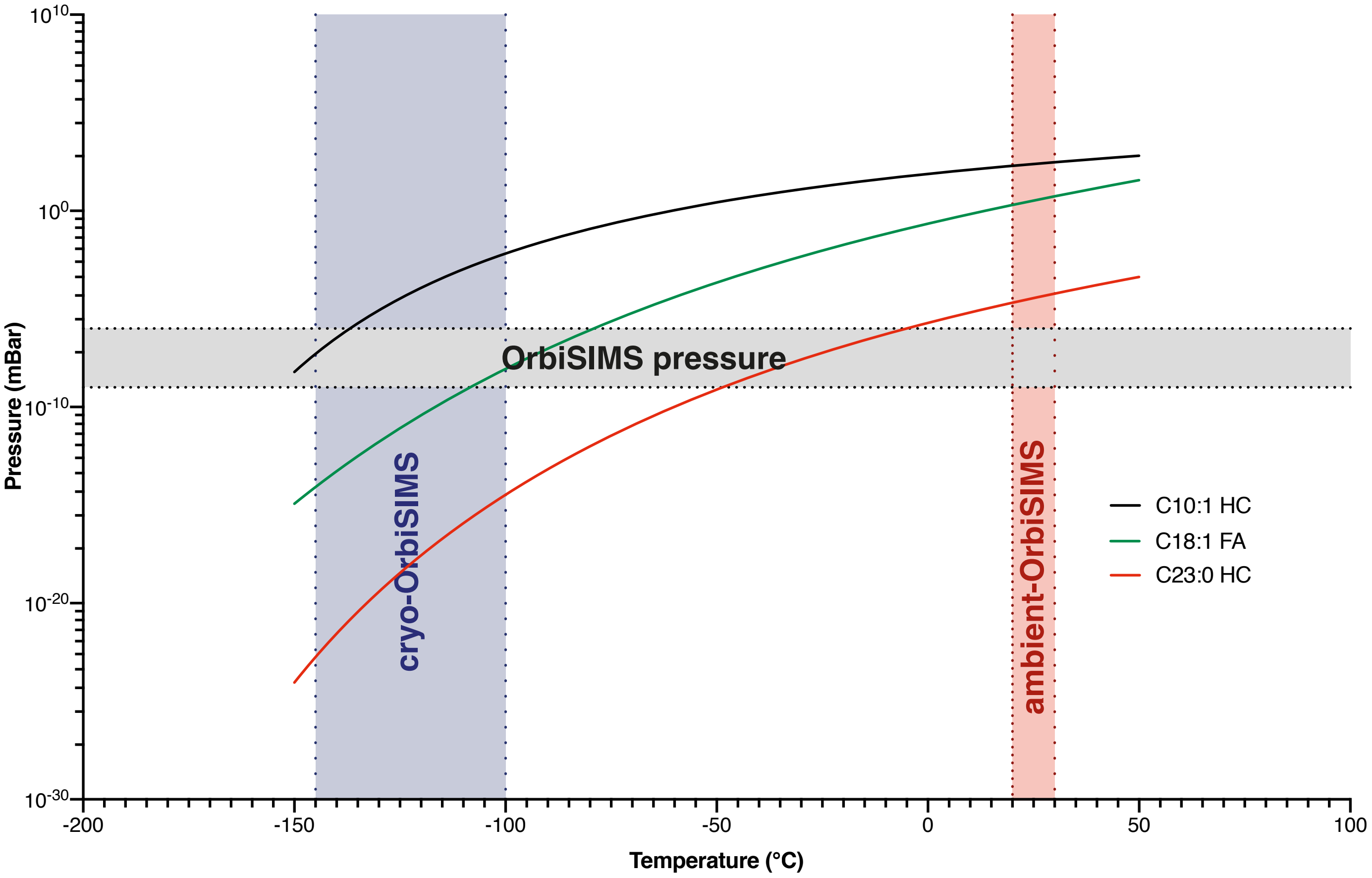

Supplementary Figure 1: Vapor pressure diagram for semi-volatile lipids

Curves indicate the predicted pressure and temperature relationship for the vaporisation of two hydrocarbons (C10:1 HC, C23:0 HC) and for oleic acid (C18:1 FA). The standard operating pressure of the OrbiSIMS is indicated at the lower end of the operating range (shaded grey area) with the higher values corresponding to increased pressures using neutral argon gas flooding. The temperature operating ranges of the OrbiSIMS in ambient (red shaded area) and cryogenic (blue shaded area) modes are indicated. Vaporization curves were calculated using the Antoine Equation (indicated) with constants from the NIST database. P = Pressure (bar) and T = Temperature (Kelvin). Constants used: C23:0 HC A=6.55706, B=4200.069 C=1.864, C18:1 FA A=12.947 B=5380.57 C=51.111, and C10:1 HC A=3.99063 B=1152.971 C=-47.301. Abbreviations: hydrocarbon (HC), fatty acid (FA).

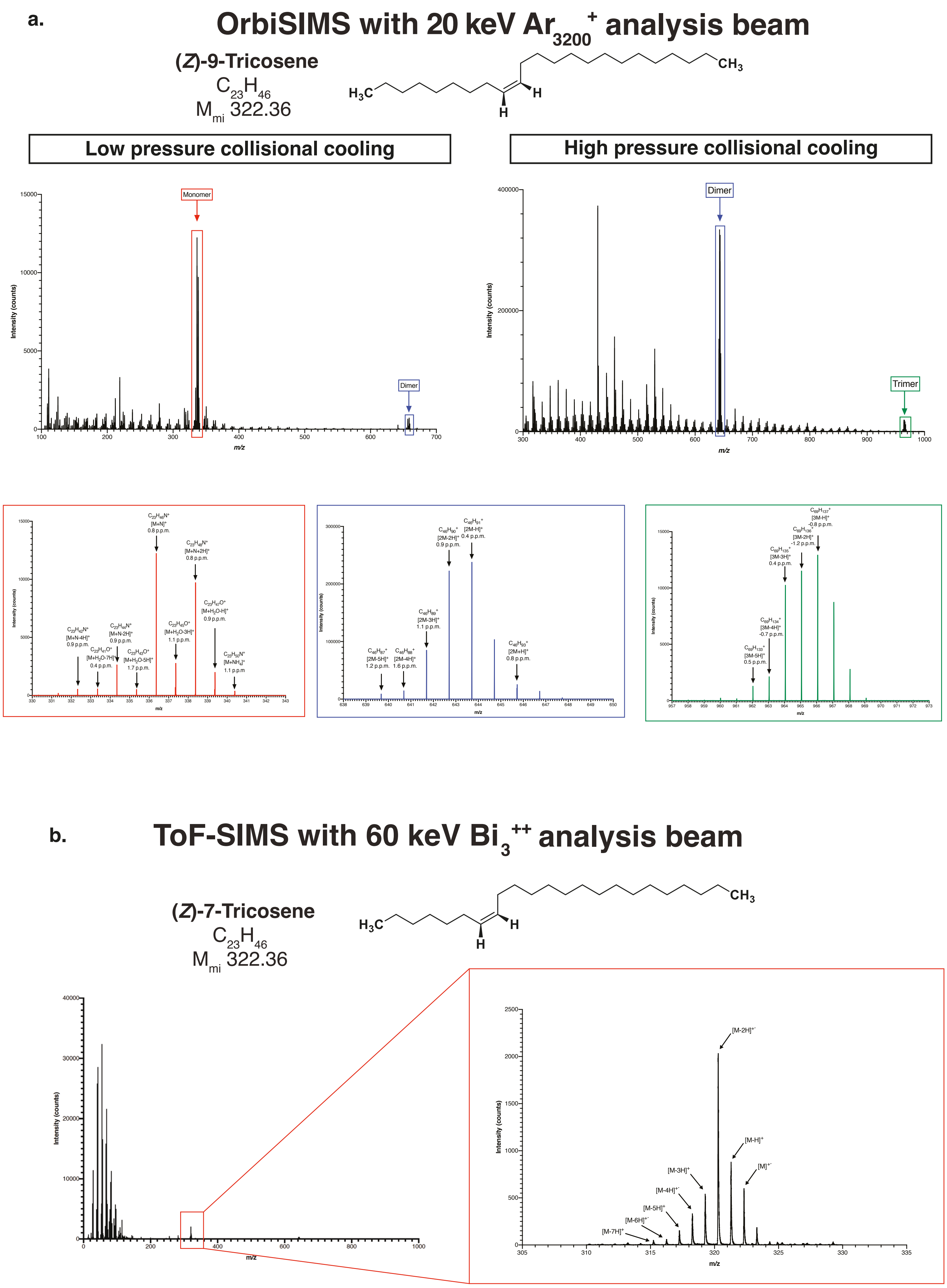

a.
OrbiSIMS with 20 keV Ar3200+ analysis beam
(Z)-9-Tricosene
C23H46
Mmi 322.36
H3C
CH3
H
H
Low pressure collisional cooling
High pressure collisional cooling
Monomer
Dimer
Dimer
Trimer
Intensity (counts)
m/z
C23H46N+
[M+N]+
0.8 p.p.m.
C23H48N+
[M+N+2H]+
0.8 p.p.m.
C46H90+
[2M-2H]+
0.9 p.p.m.
C46H91+
[2M-H]+
0.4 p.p.m.
C46H89+
[2M-3H]+
1.1 p.p.m.
C46H93+
[2M+H]+
0.8 p.p.m.
C69H137+
[3M-H]+
-0.8 p.p.m.
C69H136+
[3M-2H]+
-1.2 p.p.m.
C69H135+
[3M-3H]+
0.4 p.p.m.
C69H134+
[3M-4H]+
-0.7 p.p.m.
C69H133+
[3M-5H]+
0.5 p.p.m.
b.
ToF-SIMS with 60 keV Bi3++ analysis beam
(Z)-7-Tricosene
C23H46
Mmi 322.36
H3C
CH3
H
H
Intensity (counts)
m/z
[M-2H]+·
[M-H]+
[M]+·
[M-3H]+
[M-4H]+·
[M-5H]+
[M-6H]+·
[M-7H]+

Supplementary Figure 2: Tricosene adducts

a. Cryogenic OrbiSIMS GCIB Orbitrap analysis of the hydrocarbon (*Z*)-9-tricosene using a 20 keV Ar_{3200}^+ analysis beam with a spot size of ~3 μm summed over a 400 x 400 μm field of view with 20 x 20 pixels. The most abundant adduct incorporates nitrogen, $[M+N]^+$ and is putatively annotated as a cation. We cannot, however, rule out the possibility this may instead be a radical cation. (*Z*)-9-tricosene also forms dimers and trimers with the predominant adducts likely corresponding to $[2M-H]^+$ and $[3M-H]^+$. High and low collisional cooling pressures correspond to 1.2 x 10^{-1} mBar and 4.2 x 10-2 mBar respectively.

b. Cryogenic IONTOF 5 LMIG-ToF analysis of (*Z*)-7-tricosene using a 60 keV Bi_3^{++} analysis beam with a spot size of ~0.5 μm summed over a 500 x 500 μm field of view with 128 x 128 pixels. Extensive fragmentation is observed, although the molecular ion remains detectable. The predominant adduct likely corresponds to $[M-2H]^{+\cdot}$.

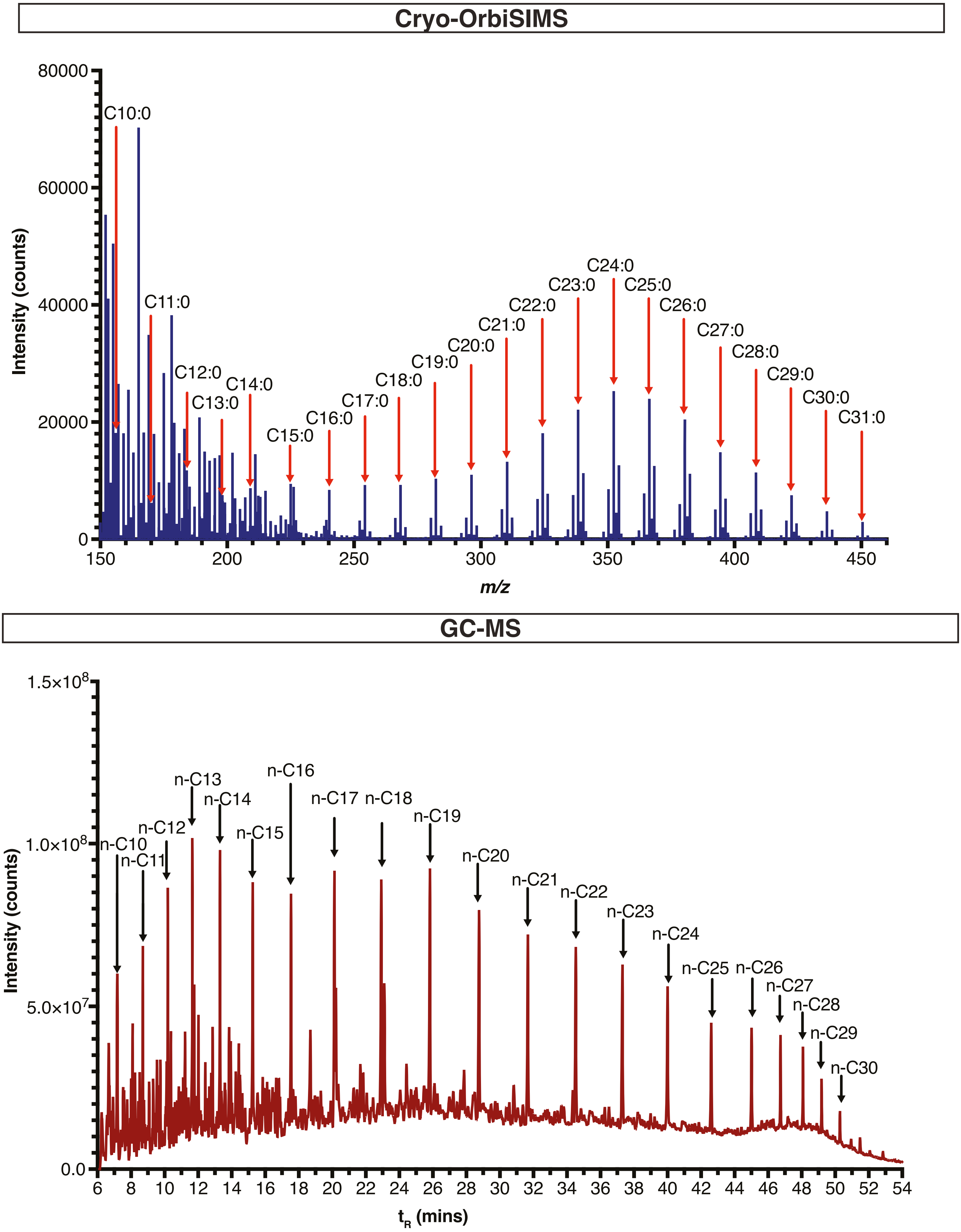

Supplementary Figure 3: Analysis of gas oil with cryo-OrbiSIMS and GC-MS
Cryo-OrbiSIMS analysis of ASTM reference gas oil identifies a wide variety of alkanes that are also detected with GC-MS. Cryo-OrbiSIMS analysis uses a 20 keV Ar_{3500}^{+} GCIB with a spot size of ~3 μm summed over a 400 x 400 μm field of view with 20 x 20 pixels.

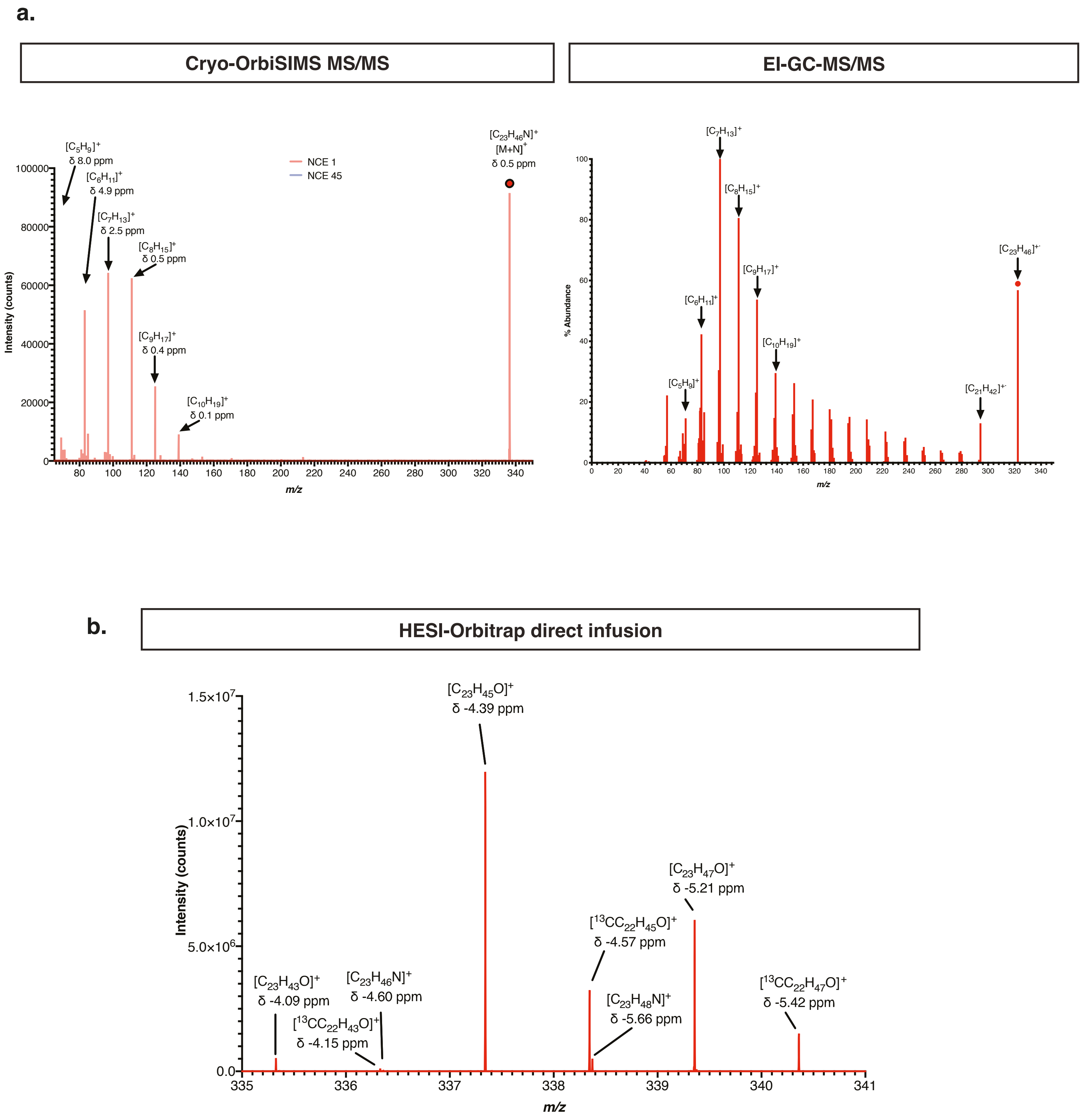

Supplementary Figure 4: Comparison of (*Z*)-9-tricosene adducts with three different MS methods

a. Cryo-OrbiSIMS MS/MS analysis of the $[M+N]^+$ tricosene adduct reveals fragmentation consistent with a linear hydrocarbon. Increasing the normalized collision energy (NCE) from 1 eV to 45 eV yields complete fragmentation of the parent ion into smaller ions. EI-GC-MS/MS analysis of the $[M]^{+\cdot}$ ion of (*Z*)-9-tricosene at a NCE of 0.5 eV yields many of the same fragments obtained via cryo-OrbiSIMS MS/MS. Cryo-OrbiSIMS analysis uses a 20 keV Ar_{3500}^+ GCIB with a spot size of ~3 μm summed over a 400 x 400 μm field of view with 20 x 20 pixels.

b. HESI-Orbitrap direct infusion of (*Z*)-9-tricosene. The most abundant adducts may result from interactions with water but nitrogen adducts, also seen with cryo-OrbiSIMS, are visible at lower abundance.

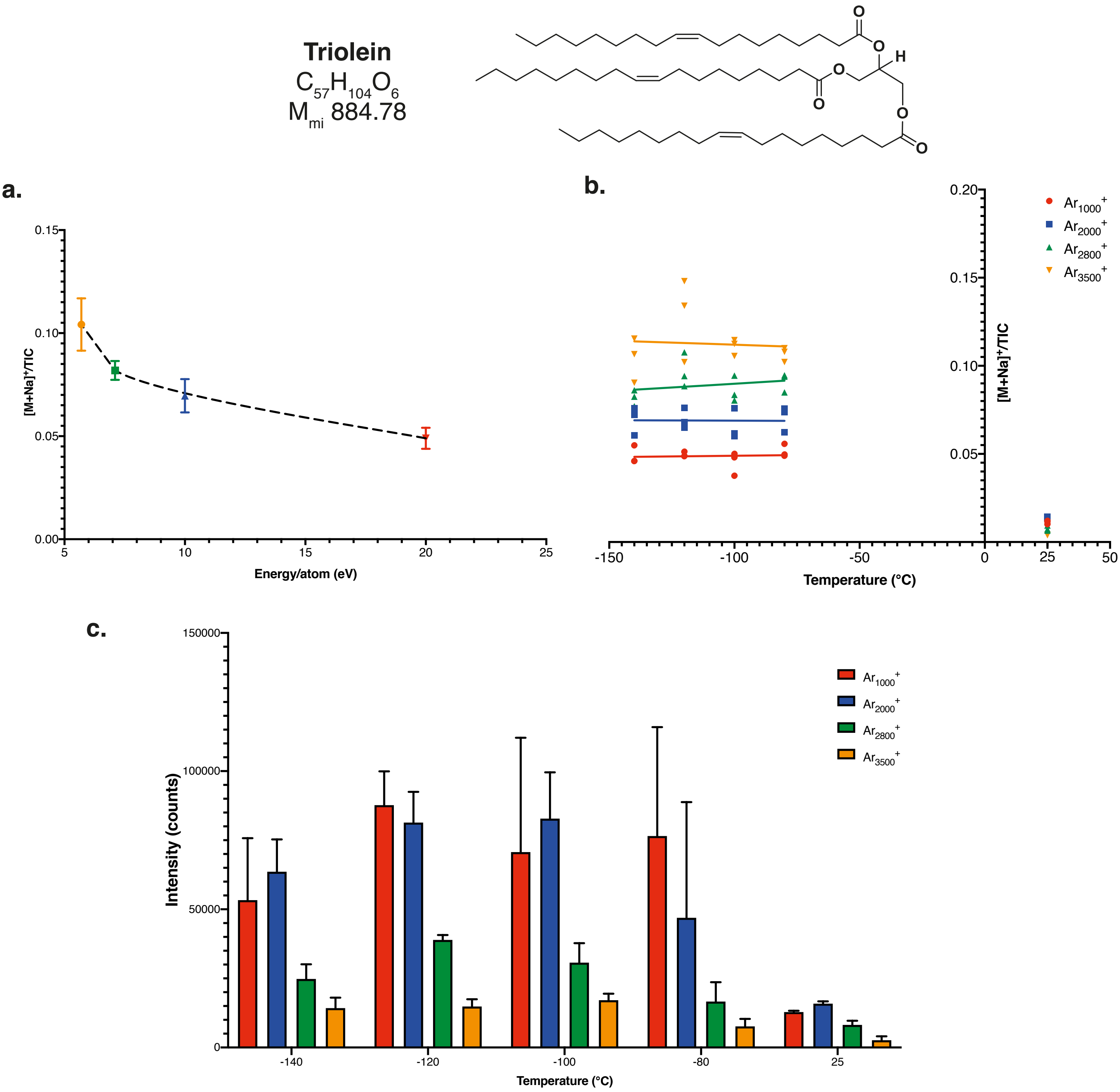

Supplementary Figure 5: Cryogenic analysis increases signal and decreases fragmentation of a TAG standard

Spectra for the TAG standard triolein were collected at the indicated temperatures using the IONTOF 5 with the ToF mass analyzer and a 20 keV Ar_n GCIB using the cluster sizes indicated summed over a 100 x 100 μm field of view with 8 x 8 pixels .

a. Ratio of $[M+Na]^+$ (quasi-molecular ion) to total ion intensity of all triolein fragments (TIC), for four argon cluster sizes from 1000 to 3500 at an analysis temperature of -140°C.

b. Ratio of $[M+Na]^+$ (quasi-molecular ion) to total ion intensity of all triolein fragments (TIC) for the agron cluster sizes and analysis temperatures indicated. Cryogenic analysis decreases fragmentation by up to ~12 fold, depending on argon cluser size.

c. Absolute signal intensity (counts) of the $[M+Na]^+$ quasi-molecular ion for the argon cluster sizes and analysis temperatures indicated. Cryogenic analysis increases the signal intensity of the quasi-molecular ion by a factor of ~4.5 for all four argon cluster sizes.

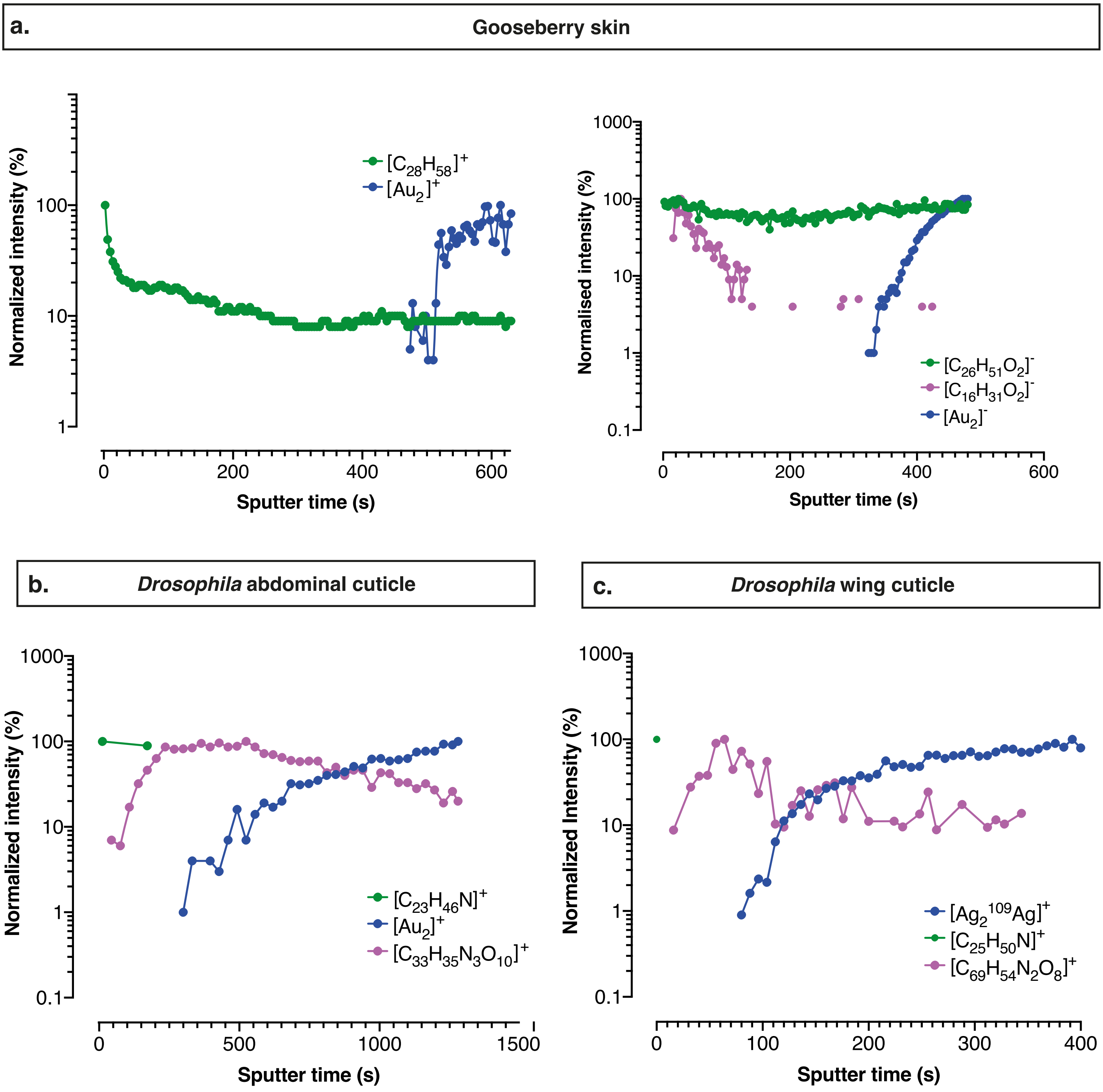
a.
Gooseberry skin
Normalized intensity (%)
Sputter time (s)
[C28H58]+
[Au2]+
Normalised intensity (%)
Sputter time (s)
[C26H51O2]-
[C16H31O2]-
[Au2]-
b.
Drosophila abdominal cuticle
Normalized intensity (%)
Sputter time (s)
[C23H46N]+
[Au2]+
[C33H35N3O10]+
c.
Drosophila wing cuticle
Normalized Intensity (%)
Sputter time (s)
[Ag2 109Ag]+
[C25H50N]+
[C69H54N2O8]+

Supplementary Figure 6: Cryo-OrbiSIMS depth profiling of SVOCs

Cryo-OrbiSIMS depth profiling with a 20 keV Ar_{3500}^+ analysis beam with a spot size of ~20 μm summed over a 40 x 40 μm field of view with 2 x 2 pixels. All depth profiles show signal intensity normalized to 100% for each compound, plotted over sputter time in seconds (s).

a. Positive polarity (left) depth profiling through the skin of a gooseberry fruit shows that the hydrocarbon octacosane ($[C_{28}H_{58}]^+$, *m/z* 408.4564, δ -0.1 ppm) is enriched at the surface of the fruit. $[Au_2]^+$ (*m/z* 393.9326, δ 0.2 ppm) is a reference for the substrate (gold-coated silicon wafer). Negative polarity (right) depth profiling indicates the presence of a putatively identified wax ester ($[C_{26}H_{51}O_2]^-$, *m/z* 395.3895, δ 0.0 ppm) distributed throughout the sample, whereas a putative free fatty acid ($[C_{16}H_{31}O_2]^-$, *m/z* 255.2330, δ 0.0 ppm) is located just below the surface of the sample. $[Au_2]^-$ (*m/z* 393.9337, δ 0.1 ppm) is a reference for the substrate.

b. Positive polarity depth profiling through the male *Drosophila* abdominal cuticle (pleura) shows that a putative C23:1 alkene ($[C_{23}H_{46}N]^+$, *m/z* 336.3628, δ 1.0 ppm) is restricted to the surface of the cuticle. $[C_{33}H_{35}N_3O_{10}]^+$ (*m/z* 1038.3868, δ -0.2 ppm) is a ubiquitous marker of the cuticle, detected throughout the depth profile. $[Au]^+$ (*m/z* 196.9660, δ -0.2 ppm) is a reference for the substrate (gold-coated silicon wafer).

c. Positive polarity depth profiling through the male *Drosophila* wing cuticle shows the presence of a putative C25:1 alkene ($[C_{25}H_{50}N]^+$, *m/z* 364.3936, δ -0.6 ppm) restricted to the surface of the cuticle. $[C_{69}H_{54}N_2O_8]^+$ (*m/z* 1038.3868, δ -0.6 ppm) is a ubiquitous marker of the wing cuticle, detected throughout the depth profile. $[Ag_2{}^{109}Ag]^+$ (*m/z* 322.7149, δ 0.5 ppm) is a silver cluster ion that marks the silver paint used to affix the sample to the gold-coated silicon wafer substrate.

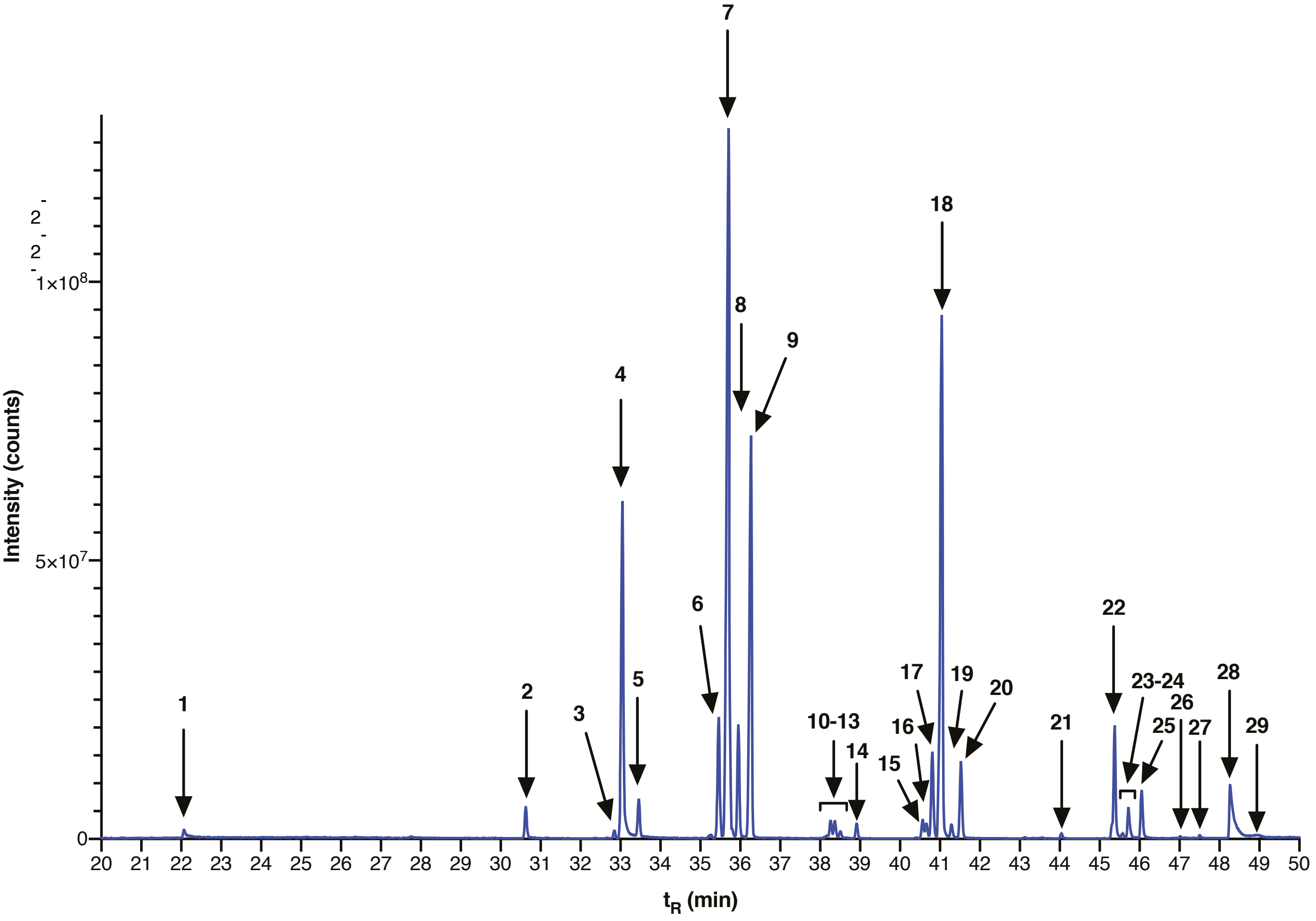

Peak number	t_R (min)	Parent ion (*m/z*)	Diagnostic ions (*m/z*)	Formula	Putative I.D.	Standard?
1	22.064	254.3	-	$C_{18}H_{38}$	octadecane	✓
2	30.626	296.3	-	$C_{21}H_{44}$	heneicosane	✓
3	308.2	308.2	-	$C_{22}H_{44}$	7-docosene	
4	33.049	310.2	250.1	$C_{20}H_{38}O_2$	11-cis-vaccenyl acetate	✓
5	33.474	310.3	-	$C_{22}H_{46}$	docosane	✓
6	35.455	322.3	-	$C_{23}H_{46}$	(Z)-9-tricosene	✓
7	35.705	322.3	-	$C_{23}H_{46}$	(Z)-7-tricosene	✓
8	35.946	322.3	-	$C_{23}H_{46}$	5-tricosene	
9	36.271	324.3	-	$C_{23}H_{48}$	tricosane	✓
10	38.165	336.3	-	$C_{24}H_{48}$	5-tetracosene	
11	38.265	336.3	-	$C_{24}H_{48}$	8-tetracosene	
12	38.365	336.3	-	$C_{24}H_{48}$	7-tetracosene	
13	38.506	336.3	-	$C_{24}H_{48}$	6-tetracosene	
14	38.918	338.3	-	$C_{24}H_{50}$	tetracosane	✓
15	40.554	352.3	337.3, 309.3	$C_{25}H_{52}$	x-methyl tetracosane	
16	40.663	348.3	-	$C_{25}H_{48}$	x,x-pentacosadiene	
17	40.804	350.3	-	$C_{25}H_{50}$	9-pentacosene	
18	41.041	350.3	-	$C_{25}H_{50}$	(Z)-7-pentacosene	✓
19	41.291	350.3	-	$C_{25}H_{50}$	5-pentacosene	
20	41.524	352.3	-	$C_{25}H_{52}$	pentacosane	✓
21	44.043	366.3	-	$C_{26}H_{54}$	hexacosane	✓
22	45.375	380.3	365.3, 337.3	$C_{26}H_{54}$	x-methyl hexacosane	
23	45.57	378.3	-	$C_{27}H_{54}$	9-heptacosene	
24	45.724	378.3	-	$C_{27}H_{54}$	7-heptacosene	
25	46.049	380.3	-	$C_{27}H_{56}$	heptacosane	
26	47.011	394.3	351.3	$C_{28}H_{58}$	x-methyl heptacosane	
27	47.502	394.4	-	$C_{28}H_{58}$	octacosane	✓
28	48.268	408.3	393.3, 365.3	$C_{29}H_{60}$	x-methyl octacosane	
29	48.955	408.3	-	$C_{29}H_{60}$	nonacosane	✓

Supplementary Figure 7: GC-MS validation of *Drosophila* cuticular hydrocarbons
GC-MS chromatogram of a hexane extract of the cuticle from five male *Drosophila* flies, showing the presence of a wide range of different hydrocarbons (as indicated in the table). The majority of these hydrocarbons are also detected by Cryo-OrbiSIMS analysis of *Drosophila* cuticle.

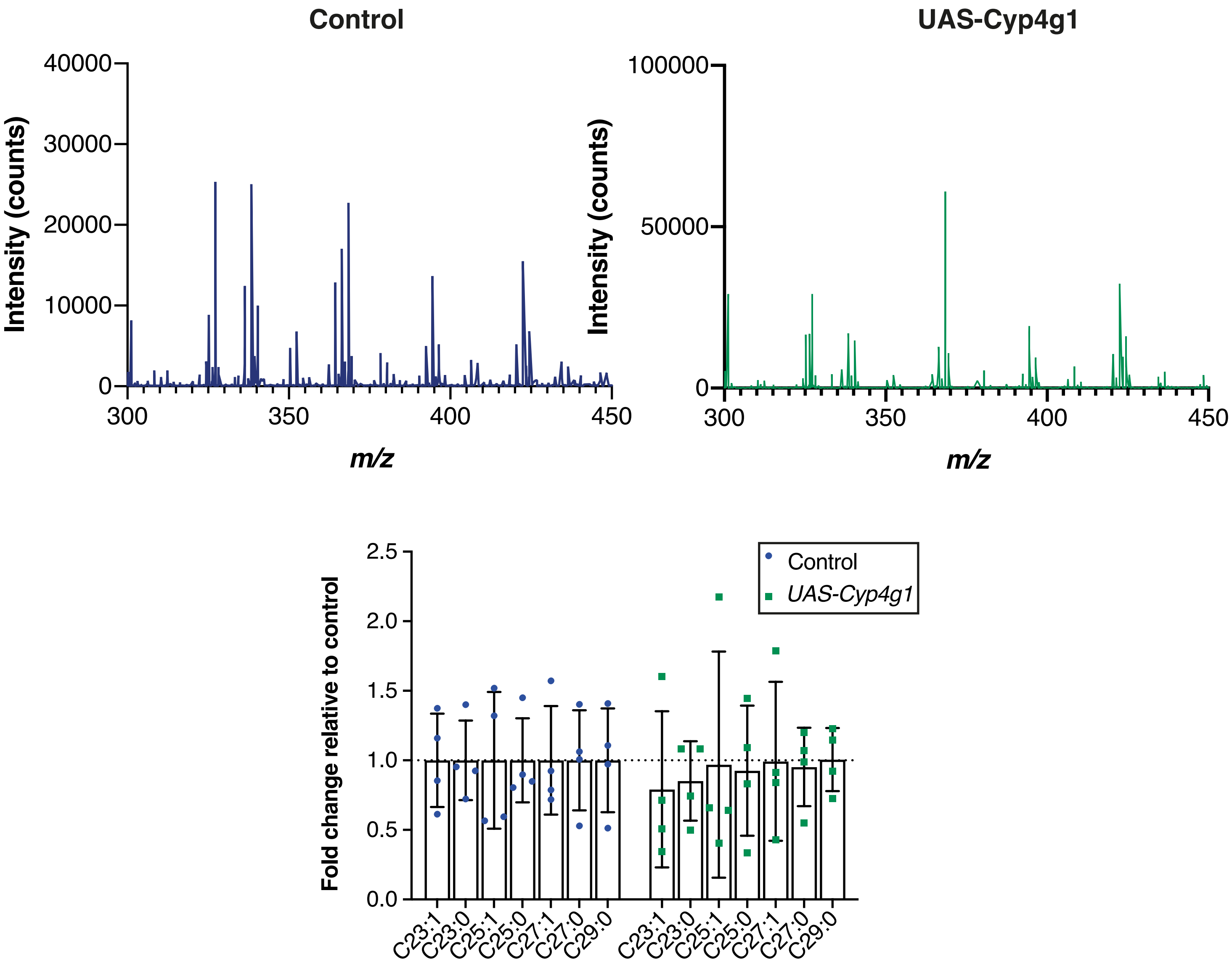

Supplementary Figure 8: Cyp4g1 overexpression does not significantly increase cuticular hydrocarbons

Cryo-OrbiSIMS Orbitrap positive polarity mass spectra using a 20 keV Ar_{3500}^{+} GCIB with a spot size of ~3 μm show *Drosophila* hydrocarbon signals are not significantly altered in response to overexpression of a hydrocarbon biosynthetic enzyme (*UAS-Cyp4g1*) - see **Supplementary Methods**. Graph shows fold changes of C_{23} to C_{29} hydrocarbons in *UAS-Cyp4g1* animals versus controls. Bars represent means, error bars are standard deviations. Cryo-OrbiSIMS measurements are summed over a 400 x 400 μm field of view with 20 x 20 pixels.

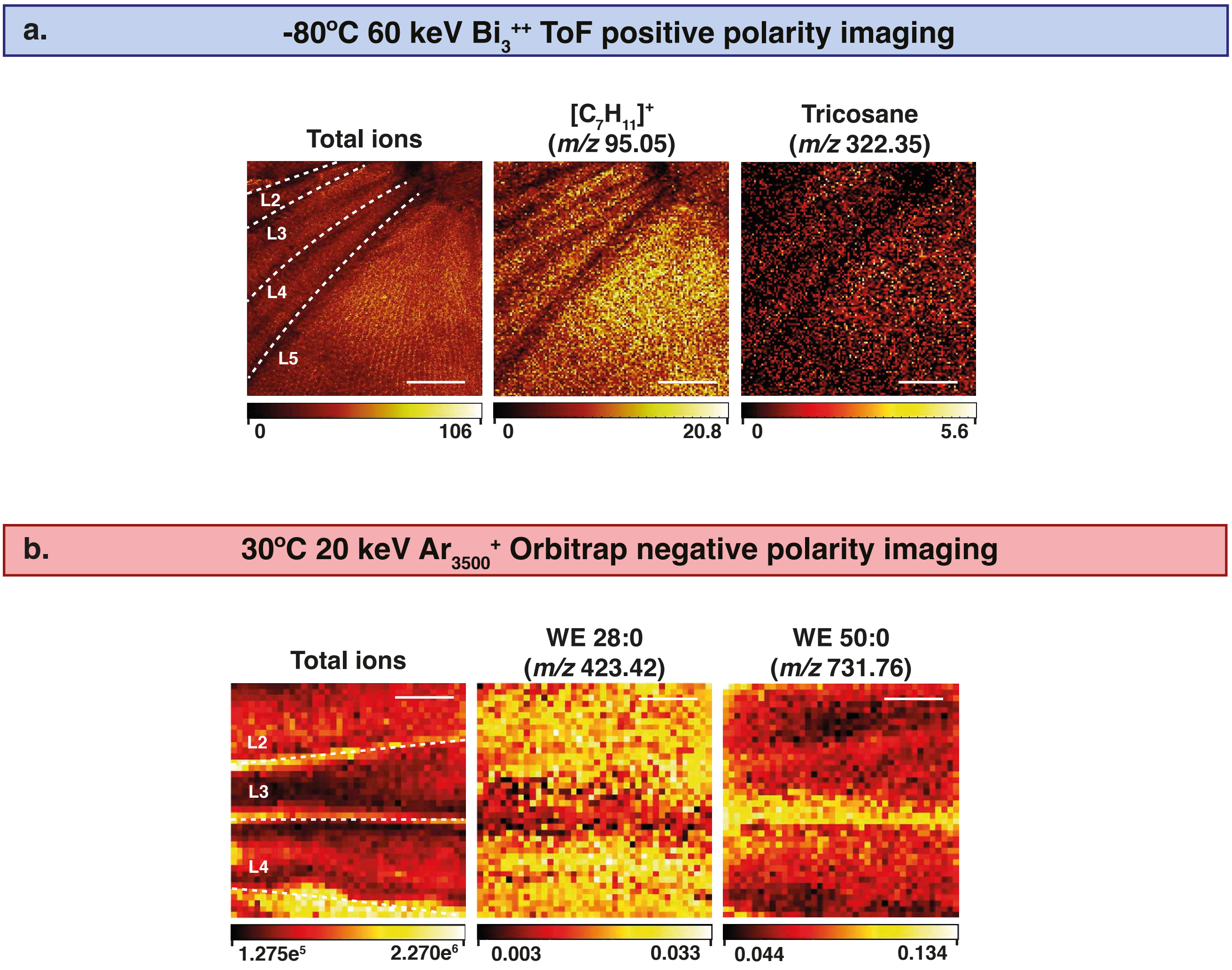

Supplementary Figure 9: Restricted localization of wax esters on the male *Drosophila* wing cuticle

a. Cryogenic ToF-SIMS imaging of a 500 x 500 μm field of view with 1024 x 1024 pixels binned to 256 x 256 pixels in positive polarity using a 60 keV Bi_3^{++} LMIG with a spot size of ~0.5 μm on the IONTOF 5. Abundant cuticular hydrocarbons are widely distributed across the wing cuticle. Putative annotations: *m/z* 322.35, $[C_{23}H_{46}]^{+}$, tricosane adduct $[M\text{-}2H]^{+\cdot}$, also detected via EI-GC/MS as $[M]^{+\cdot}$ (**Supplementary Table 1**) and confirmed with an analytical standard. *m/z* 95.05, $[C_7H_{11}]^{+}$, hydrocarbon fragment also detected in EI-GC/MS and OrbiSIMS Orbitrap MS/MS spectra of hydrocarbon standards (**Supplementary Fig. 5**). Scale bars represent 125 μm.

b. Ambient OrbiSIMS Orbitrap imaging of a 400 x 400 μm field of view with 40 x 40 pixels in negative polarity using a 20 keV Ar_{3500}^{+} GCIB wiht a spot size of ~3 μm. Putative annotations: *m/z* 423.4210, $[C_{28}H_{55}O_2]^{-}$ (mass deviation δ 0.6 ppm), C28:0 saturated wax ester (WE 28:0) adduct $[M\text{-}H]^{-}$ showing near-uniform intensity across the wing with reduced intensity surrounding the L3 vein. *m/z* 731.7656, $[C_{50}H_{99}O_2]^{-}$ (mass deviation δ 0.7 ppm), C50:0 saturated wax ester (WE 50:0) enriched on the L3 vein. *m/z* 759.7961, $[C_{52}H_{103}O_2]^{-}$ (mass deviation δ -0.3 ppm).

Wing veins are indicated on total ion images with white dotted lines and annotated below (**a**) or above (**b**) the vein.

a.

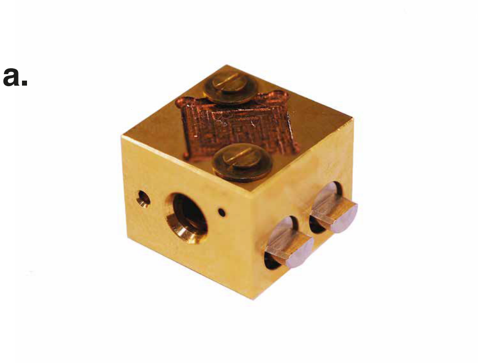

b.

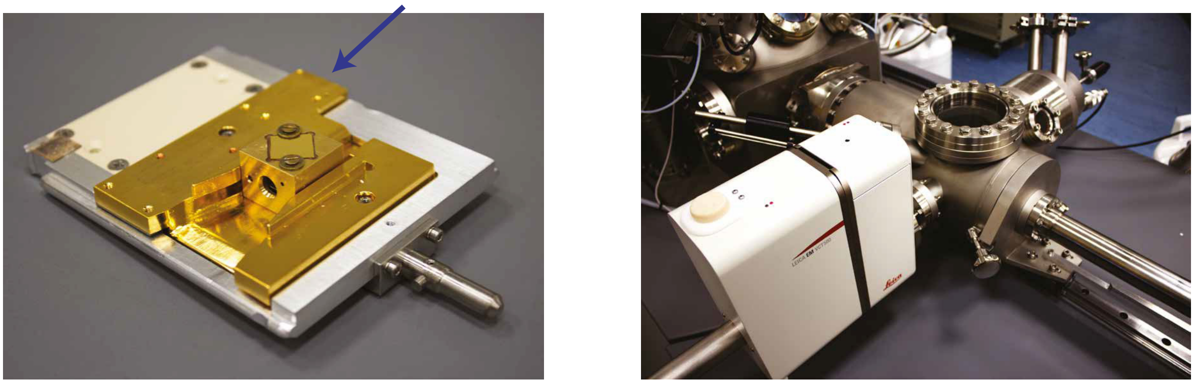

Supplementary Figure 10: Instrument modifications for Cryo-OrbiSIMS

a. Modified cryo sample holder. The sample is mounted on a conductive substrate fitting within the 10 mm x 10 mm x 0.5 mm recess. This sample holder is compatible with any SIMS instrument that accepts Leica VCT sample holders.

b. The modified sample holder is transferred under vacuum into the OrbiSIMS via a Leica EM-VCT 500 transfer arm that docks onto the instrument loadlock. The sample holder is then introduced into the IONTOF cryo-stage, which is cooled conductively in situ via a mechanically articulated copper cooling contact as indicated by the arrow.

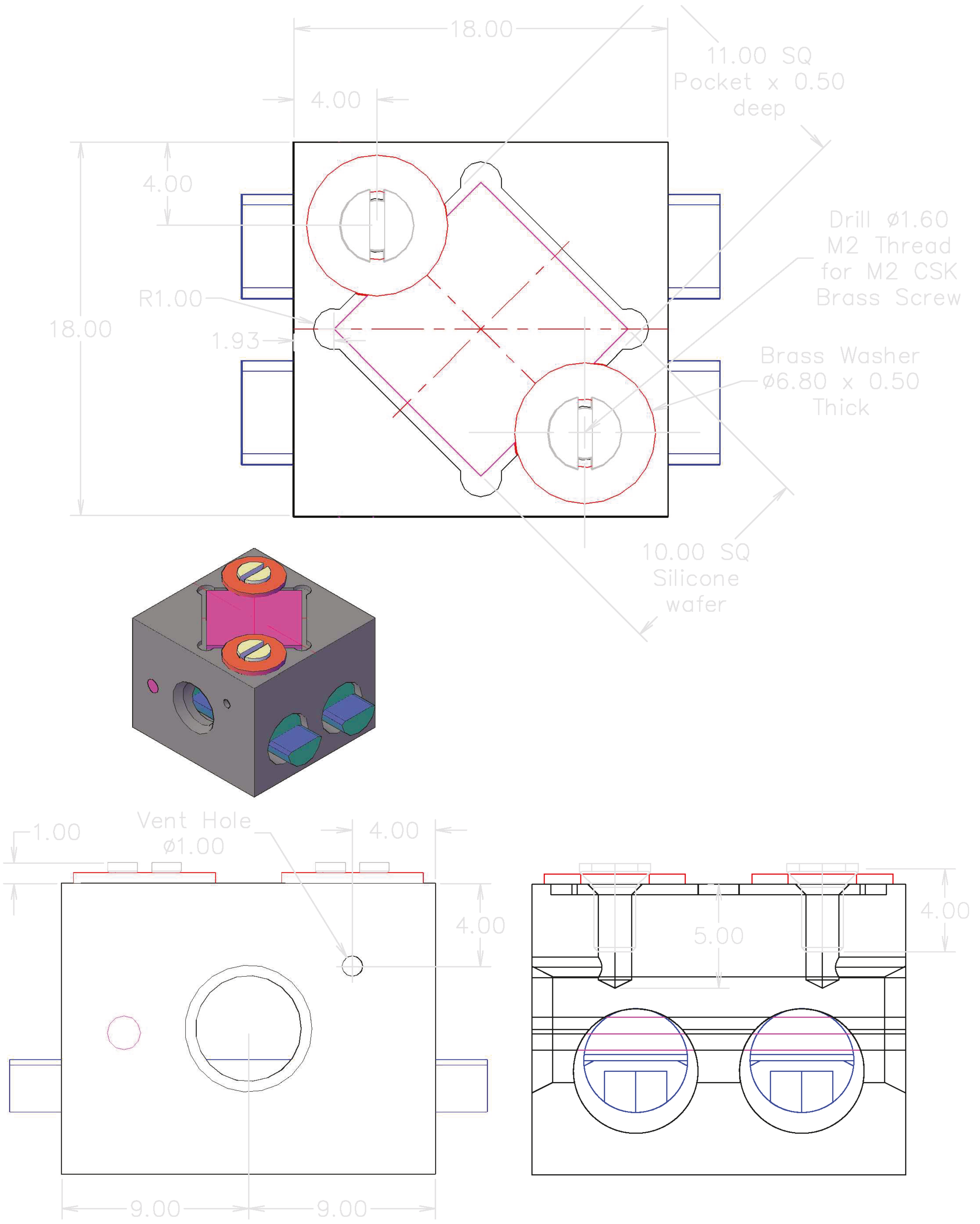

Scale 4:1

Supplementary Figure 11: Leica VCT sample holder (#16771610) modified to accommodate a 10 mm x 10 mm x 0.5 mm silicon wafer by machining a suitably sized depression into a blank sample holder. Screw threads were created and vented orthogonally to allow sample mounting under liquid nitrogen. The sample is held in place using brass washers secured with brass screws.

Experimental Section

Sample preparation

Plants: Plants were grown without the use of pesticides or herbicides. The following samples were collected: gooseberry fruit skin (*Ribes uva-crispa* cv. Hinamaki red), apple tree flower petal (*Malus domestica* cv. Bountiful), tomato leaf (*Solanum lycopersicum* cv. Sweet Millions), asparagus skin (*Asparagus officinalis* cv. Gijnlim), kiwi berry leaf (*Actinidia arguta* cv. Issai), Victoria plum (*Prunus domestica* cv. Victoria), Golden Gage plum (*Prunus domestica* cv. Oullins Golden Gage), and pine needle (*Pinus nigra*). Samples were collected, mounted on gold coated silicon wafers and stored at 4°C and analyzed within 24 hours of preparation. The leaf samples were adhered to the gold coated silicon wafer with silver conductive paint (Electrolube, #1LABLSCP/1). For GC-MS, plant samples were washed with 1 mL hexane which was dried in a fume hood. Samples were then resolubilized in 100 µL hexane.

Drosophila: All flies were raised on a standard diet composed of on a standard diet composed of: 7.5 g agar, 58.5 g glucose, 66.3 g cornmeal, 23.4 g yeast and 19.5 mL of antimycotic solution containing 0.04% Bavistan and 10% Nipagin per litre. For initial experiments, isogenic male *Drosophila* of the w1118 iso31 strain were used. Flies were raised at 25°C throughout and males separated 24 hours after eclosion and retained at 15 flies per vials until 6 days of age. For oenocyte-specific genetic manipulations, the following strains were used: PromE(800)-GAL4, tub-GAL80^{TS}, UAS-CD8::GFP/Cyo, Dfd::YFP[1] and UAS-Cyp4g1 RNAi.[2] For Cyp4g1 RNAi knockdown, flies were raised at 18°C during development and then transferred to 29°C upon eclosion. Flies were then separated by sex and genotype after 24 hours then retained at 29°C at a density of 15 flies per vial until 6 days of age. 6-day old adult male *Drosophila melanogaster* were used for OrbiSIMS experiments. Flies were anaesthetized with CO_2 and dissected in PBS to remove all tissues attached to the cuticle. Dissected cuticles were then washed with deionized water before mounting on cleaned silicon wafers (Agar Scientific # AGG3390-10) coated with gold. Samples were stored at 4°C, transported on ice and analyzed within 24 hours of preparation. For GC-MS analysis, flies were anaesthetized with CO2 and the surfaces of five flies were extracted with 50 µL hexane containing an internal standard of 0.1mM octadecane for 2 minutes in glass GC vials. The hexane extracts were transferred to glass vials with inserts (Agilent Technologies, Cat No. 5182-0715 and 5183-2085) for immediate analysis. Dimethyl disulfide derivatization was used to determine double bond position for alkenes where no standard was available. For LC-MS/MS samples, surface lipids were extracted from male Drosophila at one week of age using 100 *µ*l 2:1 chloroform:methanol for 30 seconds in a clean GC-MS vial. For positive polarity mode, 20 flies per sample were used and, for negative polarity mode, 40 flies per sample were used.

Latent fingerprints: Fingerprints were collected from three male and three female consenting volunteers on gold-coated silicon wafers, after a fingerprint conditioning protocol as previously described.[3] Samples were anonymized and stored in a secure location at 4°C for 48 hours before analysis. This study was approved by the Crick's Human Ethics Group, project number 2019 FC4, and informed signed consent was obtained in writing from all healthy volunteer participants.

3D OrbiSIMS instrumentation and workflow

3D OrbiSIMS (IONTOF GmbH) samples were mounted for cryo-OrbiSIMS analysis (Supplementary Figure 7) using a modified a blank Leica VCT holder (Leica Microsystems GmbH, cat. #16771610, Supplementary Figure 8) to enable the mounting of silicon wafers (or other appropriate substrates) under liquid nitrogen. This modified sample holder is compatible with the 3D OrbiSIMS[4] and forward compatible with IONTOF Hybrid-SIMS (IONTOF GmbH) instruments or other systems that accept Leica EM-VCT holders. After mounting samples under liquid nitrogen in a Leica EM-VCT 500 system (Leica Microsystems GmbH), samples were transferred to the instrument loadlock using the Leica EM-VCT 500 cryogenic transfer shuttle at a temperature of about -140 °C and a pressure of 2.0 x 10^{-2} mbar and parked in the loadlock on a cryo-stage cooled by a copper cooling finger until the temperature remained stable at about -135 °C at a pressure of ~1 x 10^{-5} mbar. Samples were then transferred into the analysis chamber and the stage cooled continually during analysis with a mechanically articulated thermal contact allowing movement in the x, y, z plane and cooling to between -100 and -115 °C. Analyses were performed with a 20 keV Ar_{3200}^+ quasi-continuous GCIB analysis beam with a spot size of ~3 µm and a current of 12.6 pA at 15% duty cycle. For depth profiling experiments, the GCIB was operated with a spot size of 20 µm and a current of 0.3 nA at 10% duty cycle of a 40 x 40 µm field of view with 2 x 2 pixels. Orbitrap mass calibration was performed using silver clusters generated from a standard. Spectra were acquired using a spot size of 20 µm and represent the sum of 50 scans from an area of 400 x 400 µm and 20 x 20 pixels per scan. Ions were extracted with an extraction field of 2 keV, or 500 V for *Drosophila* samples with an extraction delay of 0.925 µs to compensate for sample topography. The Orbitrap was operated in positive-ion polarity with a mass resolution of 240,000 @ 200 m/z with an injection time of 2901 ms. Mass spectral information was acquired for the mass range 100 – 1,500 *m/z*. Approximately 144500 shots at 400 µs per cycle were accumulated in the C-trap per acquisition. For MS/MS analysis, a mass window of 0.4 *m/z* was used and the normalized collisional energy set at 1 eV NCE and 45 eV NCE as indicated. To compensate for sample charging, the analysis chamber was flooded with argon gas at a pressure of 1 x 10^{-6} mbar and a flood gun was applied during the analysis with an energy of 20 eV and a current of -10 µA. For ToF imaging experiments, the LMIG was operated using 30 keV Bi_3^+ cluster beam in short pulses mode with a current of 0.06 pA. The spectra were calibrated using reference ions of $[C]^+$, $[CH]^+$, $[CH_2]^+$ and $[CH_3]^+$. The pine needle ToF image represents a 500 x 500 µm analysis area and 1024 x 1024 pixels per scan in sawtooth raster mode, with the image showing the sum of 35 scans. The total ion dose for this acquisition was 2.75 x 109 ions with 1 shot per pixel and a cycle time of 200 µs. This image was acquired between the temperatures of -112°C and -106°C. The fingerprint ToF image was acquired using a stage scan of a 2.5 x 2.5 cm area and 1250 x 1250 pixels per scan made up of 500 x 500 µm tiles automatically stitched during acquisition, with the image showing the sum of 2 scans. The total ion dose for this acquisition was 2.02 x 109 ions with 1 shot per pixel and a cycle time of 400 µs. The image was acquired between the temperatures of -125°C and -111°C. The *Drosophila* ToF images represent a 500 x 500 µm analysis area and 1024 x 1024 pixels per scan in sawtooth raster mode, with the image showing the sum of 10 scans. The total ion dose for this image was 7.85 x 10^9 ions with 1 shot per pixel, with an LMIG current of 0.2 pA and a cycle time of 200 µs. The Drosophila Orbitrap images represent a 400 x 400 µm analysis area and 40 x 40 pixels per scan with a 10 µm pixel size in sawtooth raster mode. The images show the sum of 2 scans.

ToF-SIMS imaging

TOF-SIMS 5 (IONTOF GmbH) samples were prepared on ITO coated glass coverslips (SPI supplies #6462-AB 18 mm2, resistivity 70-100 Ω), cut to size with a diamond knife, and mounted on a cooling stage. The loadlock was flooded with nitrogen gas before cooling during pump-down. During the analysis the sample was cooled with a copper cooling finger to ~ -110ºC. The analyses were performed using a 60 keV Bi_3^{++} analysis beam with a spot size of ~200 nm and a current of 0.025pA at a cycle time of 200 µs. Secondary ions were extracted with an extraction voltage of 2 keV. Measurements were obtained from a field of view of 500 x 500 µm at a resolution of 512 x 512 pixels. Measurements shown are a sum of 20 scans. To compensate for sample charging, a flood gun was applied during analysis with an energy of 21 eV and a current of -10 µA. The total ion dose was ~8.18 x 10^7 ions/cm^2. The mass resolution of the resulting spectra was ~5,000 at *m/z* 200. Spectral mass calibration was performed in reference to the known ions $[C]^+$, $[CH]^+$, $[CH_2]^+$ and $[CH_3]^+$.

ToF-SIMS spectral acquisition

A homogenous layer of a triolein standard (Sigma-Aldrich #T7140) was drop cast on to a 10 x 10 mm silicon wafer that was then mounted on a cooling stage compatible with the TOF-SIMS 5 instrument. The loadlock was flooded with nitrogen gas prior to cooling during pump-down. For analysis, the sample was cooled with a copper cooling finger to temperatures between -140ºC and -80ºC. A 20 keV Ar_n^+ gas cluster ion beam was operated in pulsed analysis mode with a cycle time of 200 µs. Secondary ions were extracted with a voltage of 2 keV and spectra collected from a field of view of 100 x 100 µm with a resolution of 8 x 8 pixels acquired in sawtooth raster mode. Measurements shown are a sum of 2,000 scans. To compensate for sample charging, an electron flood gun was applied during analysis with an energy of 21 eV and a current of -10 µA.

GC-MS

GC-MS analysis was performed as previously described.[1] In brief, GC–MS was using an Agilent 7890B-7000C GC–MS system in EI mode. Data was acquired and analyzed using MassHunter (Version B.06.00, Agilent Technologies, Inc). Splitless injection (injection temperature 270°C) onto a 30 m + 10 m × 0.25 mm DB-5MS + DG column (J&W, Agilent Technologies) was used, with helium as the carrier gas. The initial oven temperature was 50°C (1 min), followed by temperature gradients to 150°C at 10°C/min, from 150 to 249°C at 3°C/min, from 249 to 300°C at 10°C/min with a hold time of 5 min, and from 300 to 325 °C at 10 °C/min and a hold time of 12 min. Sample running order was randomized using the MassHunter sample sequence randomizer.

HESI-Orbitrap

For HESI-Orbitrap direct infusion, (*Z*)-9-Tricosene was prepared to a concentration of 1 mM in hexane. For Orbitrap analysis, the flow rate was 200 µl/min with a nitrogen sheath gas flow rate of 15.54, mass resolving power of 140,000 at *m/z* 200 and a mass range of 200 – 1500 *m/z*. The capillary voltage was set at -0.4 V and the capillary temperature at 350 ºC. The spray voltage was set at 4.5 keV.

LC-MS/MS

The LC-MS method was adapted from a published protocol[5]. The extracts were dried and resuspended in 50 µL of a mix of 1-butanol/10 mM ammonium formate in methanol (1:1, v/v). Aliquots of 5 µL were analysed by LC-MS using a Q-EXACTIVE (Orbitrap) mass spectrometer (ThermoFisher Scientific) coupled with a Dionex UltiMate LC system (ThermoFisher Scientific) equipped with a C18 Zorbax Elipse plus column (2.1 × 100 mm, 1.8 µm, Agilent). A 20 min elution gradient of 45–55% solvent B was used, followed by a 5 min wash of 100% solvent B and 3-min re-equilibration. Solvent B was water:acetonitrile:isopropanol, 5:20:75 (v/v/v) with 10 mM ammonium formate (Optima HPLC grade, Sigma-Aldrich) and solvent A was 10 mM ammonium formate in water (Optima HPLC grade, Sigma-Aldrich). Other parameters were as follows: flow rate 600 µ l/min; column temperature 60°C; and autosampler temperature 4°C. MS was performed with positive/negative polarity switching with a HESI II probe. MS parameters were as follows: spray voltage 3.5 and 2.5 kV for positive and negative modes, respectively; probe temperature 275°C; sheath and auxiliary gases were 55 and 15 arbitrary units, respectively; and full scan range: 150–2,000 m/z with settings of AGC target and resolution as balanced and high ($3e^6$ and 70,000) respectively. Data were recorded using Xcalibur 3.0.63 software (ThermoFisher Scientific). Mass calibration was performed for both ESI polarities before analysis using the Thermo Scientific Calmix standard. To enhance calibration stability, lock-mass correction was also applied to each analytical run using ubiquitous low-mass contaminants. Data-dependent mass spectra (dd-MS) were acquired in positive and negative polarities with the following parameters: AGC target and resolution set as balanced and high ($2e^5$ and 17,500), respectively; collision energy was stepped from 10 to 20 to 30 NCE in HCD (high-energy collisional dissociation) mode; TopN was 5; the isolation window was set at 0.4 *m/z*.

Data analysis

The 3D OrbiSIMS and ToF SIMS 5 instruments were controlled using SurfaceLab 7.0 (version 7.0.106074, release SL7.0e) and SurfaceLab 6.7 respectively (IONTOF GmbH), with the 3D OrbiSIMS integrating an application programming interface (API) provided by ThermoFisher Scientific.[4] Image and spectral analyses were performed using SurfaceLab 7.0 (IONTOF GmbH), MATLAB 2016a, GraphPad Prism version 8.2.1 and Origin Pro 2018 version SR1. ToF images were processed with 16 pixels binning. *Drosophila* cuticle images (ToF and Orbitrap) were normalized to the total ion count and total intensity respectively to normalize for differences in intensity caused by topography. Chemical structures were drawn using ChemSketch (ACD Labs) version 2018.1.1. For spectral data analysis, a cut off of 5,000 counts was used and a signal-to-noise ratio (SNR) of 1,000. Mass deviation (δ in ppm) was calculated using the average observed mass over three separate measurements. ToF peak assignments were validated by overlaying ToF spectra with the corresponding high mass resolution Orbitrap spectra from the same field of view. GC-MS data analysis was performed using MassHunter Qualitative Analysis software (Version B.06.00, Agilent Technologies, Inc). Hydrocarbons were identified based on comparisons to spectra and retention times of authentic standards (indicated where available). HESI-Orbitrap data analysis was performed using ThermoFisher XCalibur SP1 Qual Browser version 4.1.50. Metabolomics data have been deposited to the EMBL-EBI MetaboLights database (DOI: 10.1093/nar/gkz1019, PMID:31691833) with the identifier MTBLS1873.The complete dataset can be accessed here: “https://www.ebi.ac.uk/metabolights/MTBLS1873".[6]

References

[1] M. I. Stefana, P. C. Driscoll, F. Obata, A. R. Pengelly, C. L. Newell, J. I. MacRae, A. P. Gould, *Nature Communications* **2017**, 8, 1384.
[2] E. Cinnamon, R. Makki, A. Sawala, L. P. Wickenberg, G. J. Blomquist, C. Tittiger, Z. e. Paroush, A. P. Gould, *PLOS Genetics* **2016**, 12, e1006154.
[3] S. Pleik, B. Spengler, T. Schäfer, D. Urbach, S. Luhn, D. Kirsch, *Journal of The American Society for Mass Spectrometry* **2016**, 27, 1565-1574.
[4] M. K. Passarelli, A. Pirkl, R. Moellers, D. Grinfeld, F. Kollmer, R. Havelund, C. F. Newman, P. S. Marshall, H. Arlinghaus, M. R. Alexander, A. West, S. Horning, E. Niehuis, A. Makarov, C. T. Dollery, I. S. Gilmore, *Nature Methods* **2017**, 14, 1175-1183.
[5] S. Amiar, J. I. MacRae, D. L. Callahan, D. Dubois, G. G. van Dooren, M. J. Shears, M.-F. Cesbron-Delauw, E. Maréchal, M. J. McConville, G. I. McFadden, Y. Yamaryo-Botté, C. Y. Botté, *PLOS Pathogens* **2016**, 12, e1005765.
[6] K. Huang, K. Cochrane, V. C. Nainala, M. Williams, J. Chang, K. V. Jayaseelan, C. O'Donovan, *Nucleic Acids Research* **2019**, 48,D440-D444.
